# Global trends in polycystic ovary syndrome research: A 10-year bibliometric analysis

**DOI:** 10.3389/fendo.2022.1027945

**Published:** 2023-01-09

**Authors:** Na Shi, Hong-bo Ma

**Affiliations:** ^1^ Department of Traditional Chinese Medicine, Shandong Provincial Hospital Affiliated to Shandong First Medical University, Jinan, China; ^2^ The First Clinical Medical College, Shandong University of Traditional Chinese Medicine, Jinan, China

**Keywords:** polycystic ovary syndrome, current status, global research trends, research hotspots, bibliometrics

## Abstract

**Background:**

Polycystic ovary syndrome (PCOS) is one of the most common reproductive metabolic disorders in women, significantly affecting the biological functionalities of ovaries. This disease has garnered enormous interest from researchers. However, there is a lack of a comprehensive research concerning assessing the current status and future trends in PCOS field. This study uses bibliometric tools to comprehensively analyze the PCOS-related research progress based on the literature in the past decade.

**Methods:**

The reported PCOS literature in the past decade is downloaded from the Web of Science database. The bibliometric software is applied to analyze the co-authorship, co-citation, and co-occurrence status.

**Results:**

A total of 9936 publications imported into bibliometric tools for analysis show a sharp increase in the annual citations. The USA is dominant in terms of contribution in the field of PCOS, while China is making a significant contribution to the advancement of this field. Monash University is the most prolific institution with the highest H-index value. The contribution of University of Adelaide must be acknowledged. Legro RS and Teede HJ are the most active and influential authors in recent times, while Azziz R is the most contributed pioneer in this field. The Journal of *Clinical Endocrinology & Metabolism* is the most active journal with the highest number of publications and citations. The pathogenesis of PCOS had been a long-term forefront of research. In recent years, the health management in PCOS prevention and long-term complications was attracting more and more attention. The keywords like “gut microbiota”, “microRNAs”, “apoptosis”, “Myo-inositol”, “TNF-alpha”, “androgen receptor”, and “Vitamin D-deficient” are considered the latest research topics.

**Conclusion:**

The study comprehensively analyzes the current status and global trends in the PCOS field, providing a significant reference for researchers to explore this field effectively.

## Highlights 

The study comprehensively analyzes the current status and global trends in the PCOS field, providing a significant reference for researchers to explore this field effectively.

## Introduction

Polycystic Ovary Syndrome (PCOS) is one of the most common heterogeneous endocrine disorders in women of reproductive age, involving endocrine, reproductive, and metabolic systems. The underlying etiology of this disease is highly complicated with the key clinical manifestations of hyperandrogenemia (clinical and/or biochemical), oligo/anovulation, and polycystic ovaries on ultrasound (1). Based on the NIH 1990 guide and Rotterdam 2003 criteria, the global prevalence rate of PCOS is in the range of 4-21% (2), in which 5.6% of the PCOS cases account for the Chinese women aged 19-45 years (3). This significant variance could be due to differences in races and diagnostic criteria. Notably, the global incidence rate of PCOS has shown a rapidly increasing trend annually along with the severe long-term complications with age, including infertility, obstetrical problems, type 2 diabetes mellitus, cardiovascular diseases, endometrial cancer, psychological problems, resulting in a deprived life quality (4–8). Considering these aspects, PCOS has garnered enormous interest from researchers in various fields at home and abroad.

In virtue of the magnitude of complications and the complexity, as well as the uncertainty of PCOS pathogenesis, enormous literature of original research and review articles have been reported concerning epidemiology, pathophysiology, diagnosis, and treatment of PCOS. Nevertheless, it is challenging to summarize and analyze the advancements in the field on a large scale by the traditional systematic reviews. To a considerable extent, bibliometric analysis has emerged as an essential research tool for quantitatively evaluating the scholarly literature and efficiently predicting the forefront hotspots in global scientific research based on mathematical models and statistical techniques (9). Although the bibliometric analysis has been widely applied in different fields of medicine, there has been only a single article on bibliometric analysis of PCOS presented data from 3 years ago (10). In an attempt to evaluate contributions to this field, a bibliometric analysis was applied to qualitatively and quantitatively analyze the latest PCOS literature published in the past decade (2012–2021) and track the trend and hotspots in the future.

## Materials and methods

### Data source and search strategy

**Figure 1:**
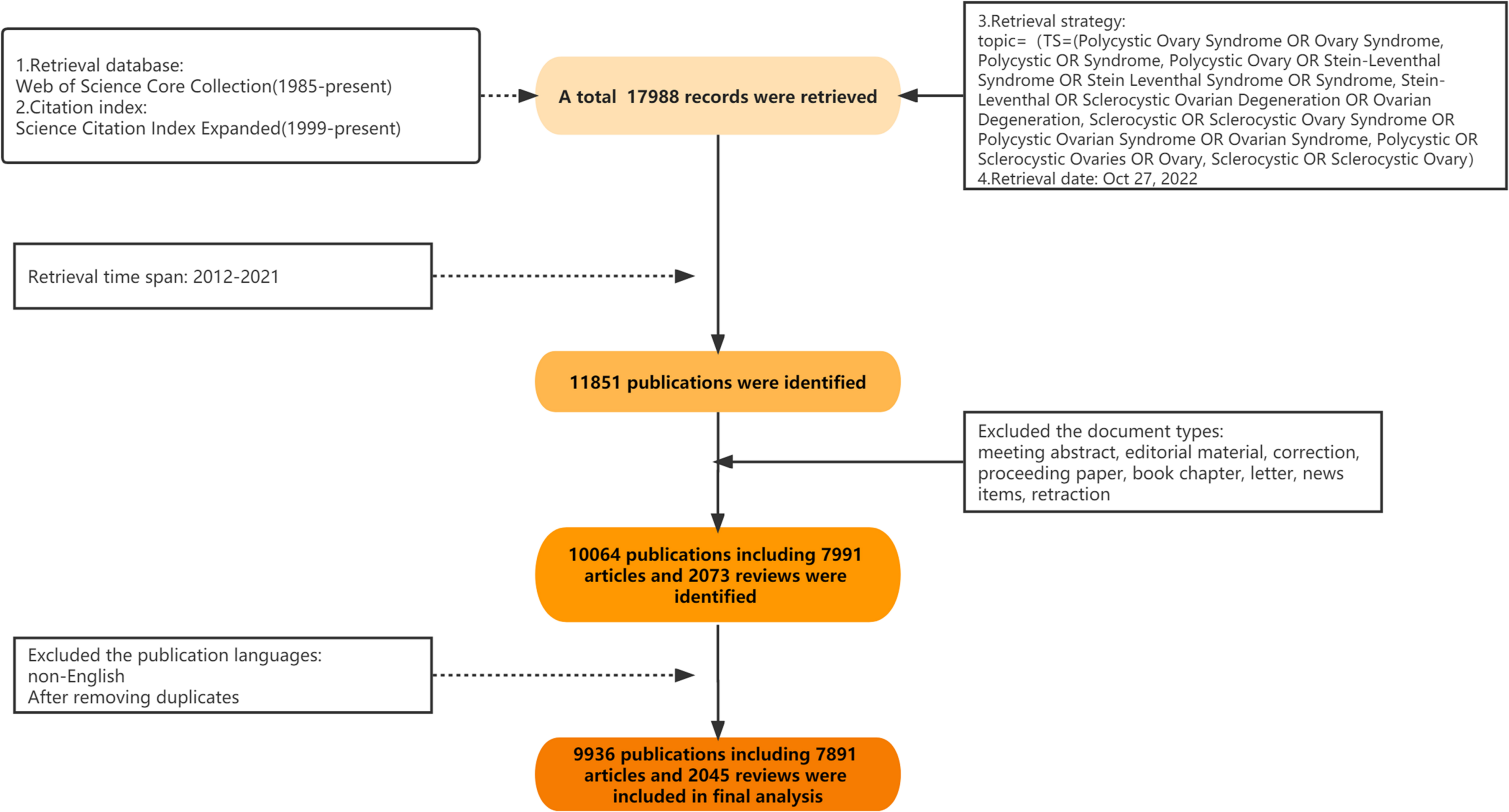
Flowchart of literature search and selection process.

The bibliometric study was conducted based on a literature search using the Web of Science Core Collection (1985-present). The original data were downloaded and extracted from the Science Citation Index Expanded database (SCIE, 1999-present, last accessed October 28, 2022). The detailed search was performed using items as follows: TS=(Polycystic Ovary Syndrome OR Ovary Syndrome, Polycystic OR Syndrome, Polycystic Ovary OR Stein-Leventhal Syndrome OR Stein Leventhal Syndrome OR Syndrome, Stein-Leventhal OR Sclerocystic Ovarian Degeneration OR Ovarian Degeneration, Sclerocystic OR Sclerocystic Ovary Syndrome OR Polycystic Ovarian Syndrome OR Ovarian Syndrome, Polycystic OR Sclerocystic Ovaries OR Ovary, Sclerocystic OR Sclerocystic Ovary), with the publication language restricted to English. The timespan for retrieval was set as 2012 to 2021, and the document type was limited to Article or Review. Notably, ethical approval was not required for this analysis as all the data used in this study were downloaded from the public database, and no human participants were involved. A flowchart illustrating the literature search and selection process is displayed in **Figure 1**.

### Data extraction and collection

According to the aforementioned search criteria, all records of qualified literature were initially downloaded as a plain text format. Because CiteSpace could only identify the files named with “download_*.txt”, all files needed to be renamed and placed in a folder named “input”. Then, the files were imported into CiteSpace 5.8.R3 (Chaomei Chen, Drexel University, USA) for removing duplicates. Further, the duplicate reports were identified and removed, and all data were manually inspected thrice to ensure the accuracy of the results. Using the “citation report” function in Web of Science, the total number of citations, average citations of each item (ACI), and Hirsch index (H-index) were obtained. ACI and H-index are the two important indicators to measure the impact of individuals, publications, or institutions. The impact factor (IF) and Journal Citation Reports (JCR) category quartile rankings (Q1–Q4) of journals were extracted from the online journals retrieval platform (https://www.medsci.cn/sci/index.do).

### Bibliometric analysis

In this study, the CiteSpace (version 5.8.R3) (11) and VOSviewer (version 1.6.18) (12) and a free online tool of literature analysis (https://bibliometric.com/) were applied in the bibliometric data analysis and network visualization due to their complementary advantages. Throughout the process of data visualization, some data needed to be cleaned or summarized. For example, the data from Taiwan or Hong Kong was pooled into China, and the data from England, Northern Ireland, Scotland, and Wales was merged into the United Kingdom. In the keyword analysis, some similar keywords were merged into one.

CiteSpace, a Java-based software, was developed by Professor Chaomei Chen for information visualization. This software was used to establish the cooperation relationships of institutions, the co-occurring network for subject categories, co-citation analysis and dual-map overlay analysis for journals, and bursts analysis for references and keywords. In the parameters settings of CiteSpace, the time slice was set as one-year, and the note type was selected as author, institution, country, keyword, and reference, respectively. In addition, the Top N per slice was set as 50. The color-coded nodes and edges were created in the network, with a different color assigned to each year. The high degree of betweenness centrality (BC≥0.1) was represented by a purple ring in a node, indicating the number of links of one node relative to another (13). A dual-map overlay analysis in CiteSpace could provide the citing trajectories displaying the dynamics of previous cross-discipline study activities (14).

The VOSviewer, a software tool for constructing and visualizing bibliometric networks, was developed by the Centre for Science and Technology Studies at the Leiden University in Leiden, the Netherlands. By setting the two parameters of “Create a map based on bibliographic data” and “Read data from bibliographic database files”, this software tool was used for the following visual analyses of co-authorship of countries/regions, institutions, and authors, co-citation of authors, and co-occurrence of keywords. The visualizing networks were generated in VOSviewer. The total link strength (TLS) is a valuable parameter applied in the VOSviewer to measure the strength of associations among countries, authors, journals, and keywords. It should be noted that the greater the TLS, the higher the cooperation or co-occurrence of the two notes (15).

In this study, the online tool of literature analysis (https://bibliometric.com/) was used to analyze the cooperation among countries/regions. The downloaded data from Web of Science database was used as the data source.

In addition, an online mapping platform (http://www.bioinformatics.com.cn/) was used to produce the world map displaying the contribution of each country, and Microsoft Excel v2016 were applied to for data collection and plotting graphs.

## Results 

### Analysis of global trends of publications and citations

The number of publications and citations are the essential indicators of the development trends in this bibliometric analysis. Overall, 9936 publications met the filtering criteria to be imported into bibliometric tools for subsequent analysis, including 7891 original articles and 2045 reviews. Similarly, annual citations also showed a sharp increasing trend from 548 in 2012 to 47038 by 2021 (**Supplementary Figure S1**).

### Analysis of the contributions of counties/regions

**Figure 2:**
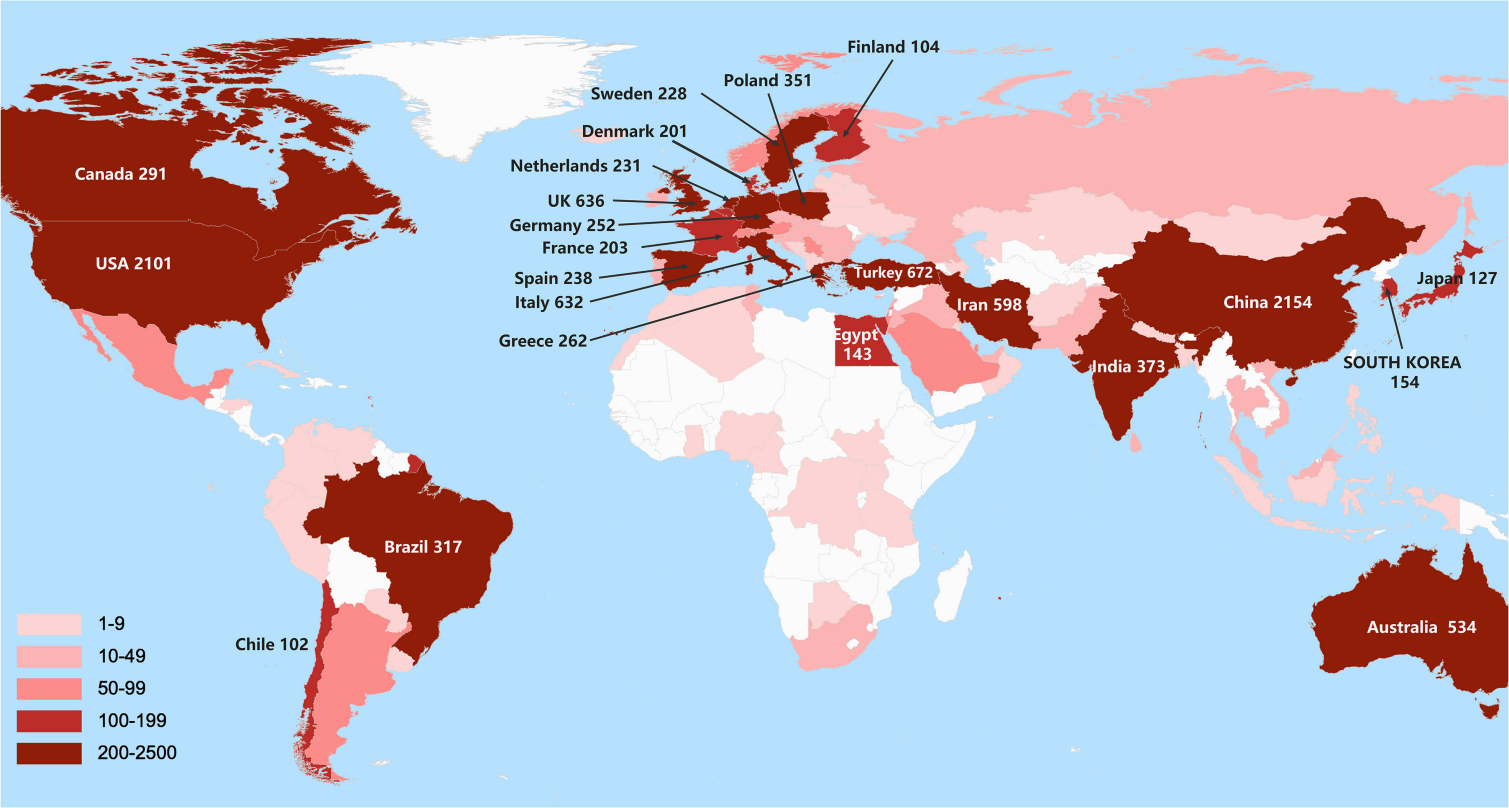
A world map displaying the contribution of each country to PCOS research based on publication counts: the darker the color, the more publications, as shown at the bottom left.

Notably, all the reported publications related to PCOS research were distributed among 140 countries/regions (**Figure 2**). The top 20 high-yield countries with H-index are listed in **Supplementary Table S1**. Among various countries, the highest number of publications was reported from China with 2154 articles, followed by the USA with 2101, while the rest have published less than 700 articles. From the perspective of the H-index, the countries with the highest indices were the USA (114), the UK (72), Australia (69), Italy (66) and China (63).

**Supplementary Figures S2**-**S3** show the visualization of cooperative relationships among countries/regions. Similar to H-index, the top five active countries with the greatest TLS included the USA (1351 times), the UK (940 times), Italy (636 times), Australia (618 times), and China (571 times). Among these top five active countries list, the USA, the UK and China were most closely related. In addition, the USA, Canada, Australia, and India demonstrated active collaborations, while the UK also showed close cooperation with Qatar and the Netherlands. From the co-authorship overlay visualization map from VOSviewer, the green and yellow nodes presented that the average appearing years were later than others, with the representative countries including China, Iran, India, Australia, and Poland.

### Analysis of the productiveness and co-authorship of institutions

**Figure 3:**
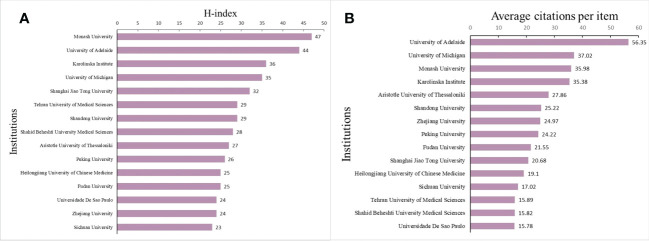
**(A)** The top 15 institutions according to H-index. **(B)** The top 15 institutions according to ACI.

The top 15 institutions with the highest number of publications are listed in **Supplementary Figure S4**. Among them, Monash University was the most productive institution with 241 articles, followed by Shanghai Jiao Tong University (231 articles), Tehran University of Medical Sciences (152 articles), and Shandong University (149 articles). Moreover, in terms of the H-index, as presented in **Figure 3A**, Monash University (47), University of Adelaide (44), and Karolinska Institute (16) were stood in the top three ranks in the list. As the second most published institution, Shanghai Jiao Tong University showed an H-index of only 32. While the publication of University of Adelaide was not many (148 articles), its H-index was in the top ranks. The top 15 institutions in terms of ACI are shown in **Figure 3B**. University of Adelaide significantly outranked other institutions working in this field with the highest ACI (56.35), followed by University of Michigan (37.02), Monash University (35.98) and Karolinska Institute (35.38).

**Figure 4:**
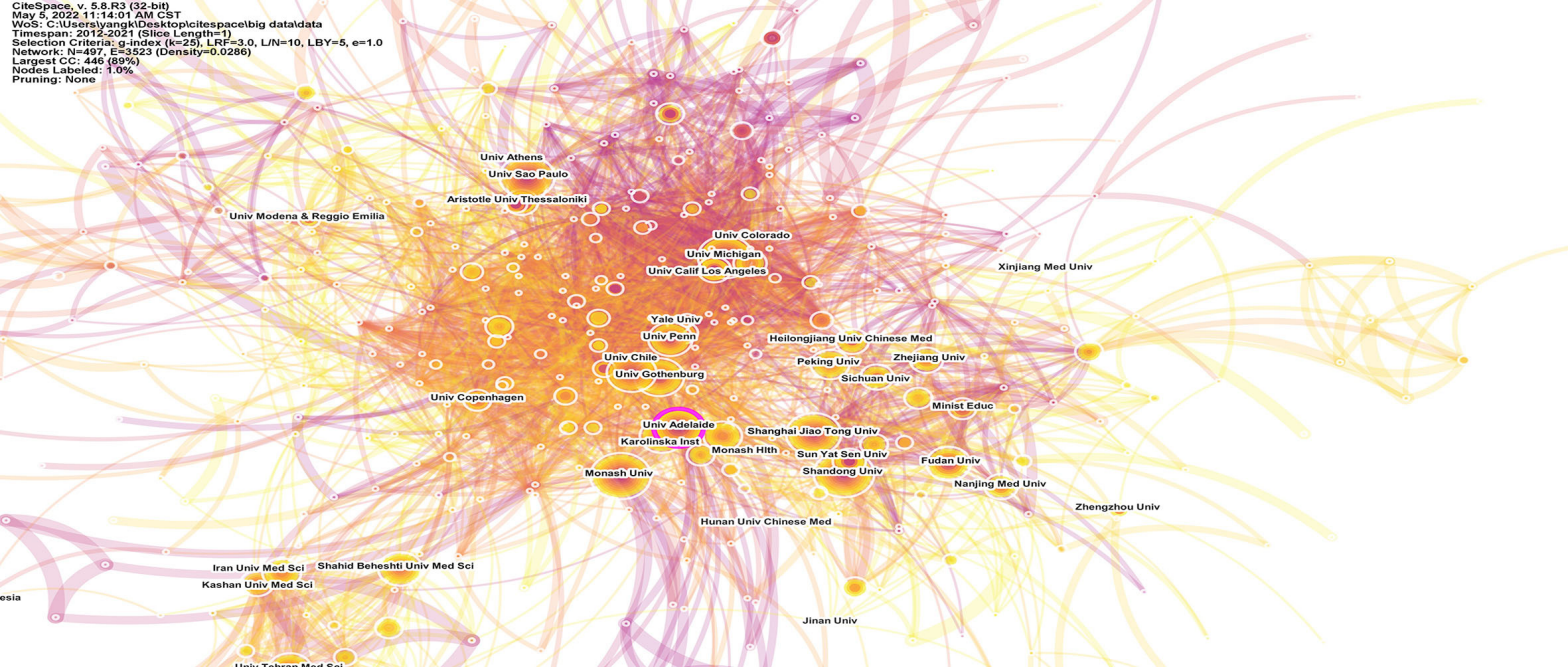
The network map of institutions by Citespace. The node size represents the number of publications from the institution. Node with purple ring in the network reflects it with high betweenness centrality (BC ≥ 0.1).

**Figure 5:**
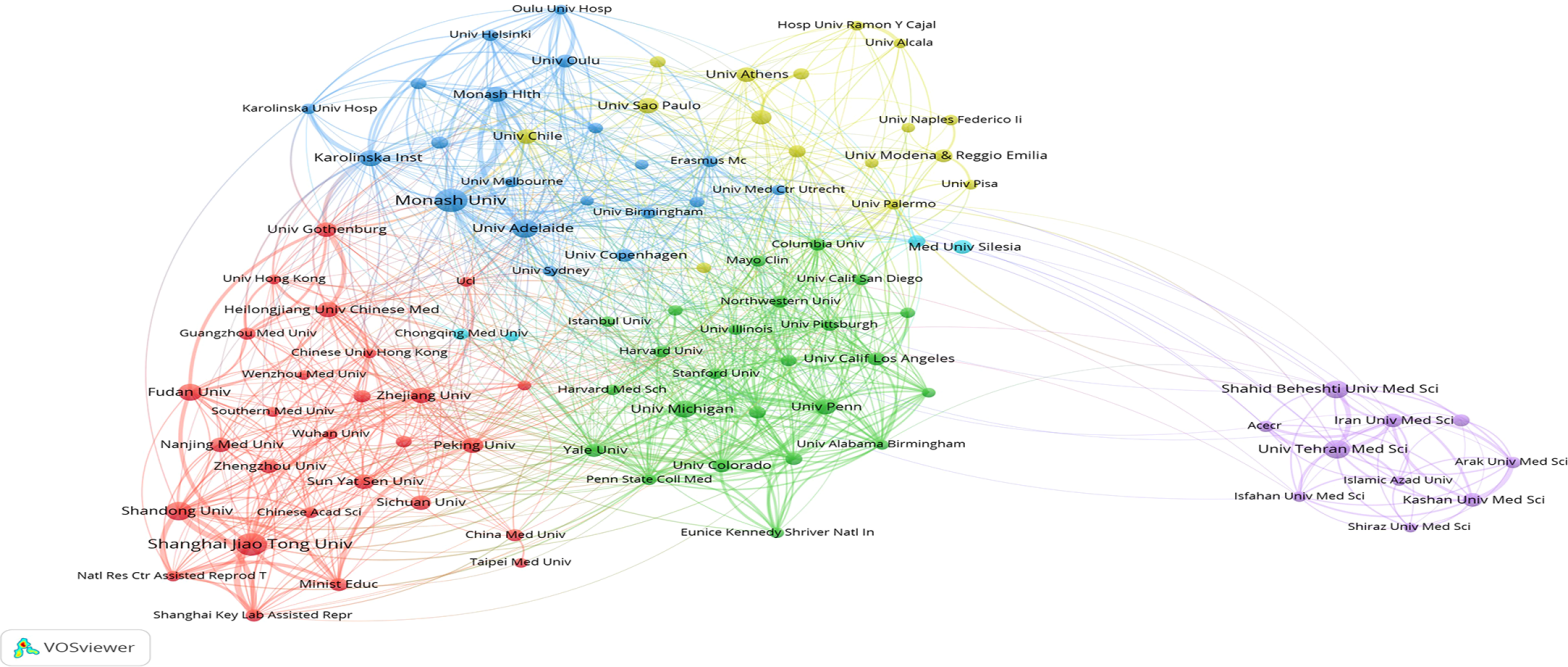
Institution co-authorship network visualization map by VOSviewer. The distance between two nodes indicates the relatedness of institutions in terms of co-authorship links. The smaller distance, the stronger relatedness, defined as one cluster with the same color.

The co-authorship relationship among institutions was performed using CiteSpace software (**Figure 4**) and VOSviewer (**Figure 5**). It was observed that University of Adelaide was the only institution with a higher BC value than 0.1 in the purple ring and identified as a critical network hub connecting many institutions, suggesting no substantial influence on other institutions. In this aspect, three significant clusters were clearly identified to dominate the research domain in the co-authorship network map, predominantly concentrated in institutions from Australia, USA, and China, respectively. In addition, many institutions from Iran formed a tight cluster, which was indistinctly placed from the significant clusters. The top 3 institutions with the greatest TLS included Monash University (715), University of Adelaide (498), and University of Pennsylvania (480).

### Analysis of funding agencies

The top 15 worldwide funding agencies for the support of PCOS research are summarized in **Supplementary Figure S5**. Six funding agencies (46% of all studies) were from the USA. National Institutes of Health ranked first, supporting 1020 studies, and the National Natural Science Foundation of China was indicated second, sponsoring 939 studies.

### Analysis of productiveness and co-authorship of authors

**Figure 6:**
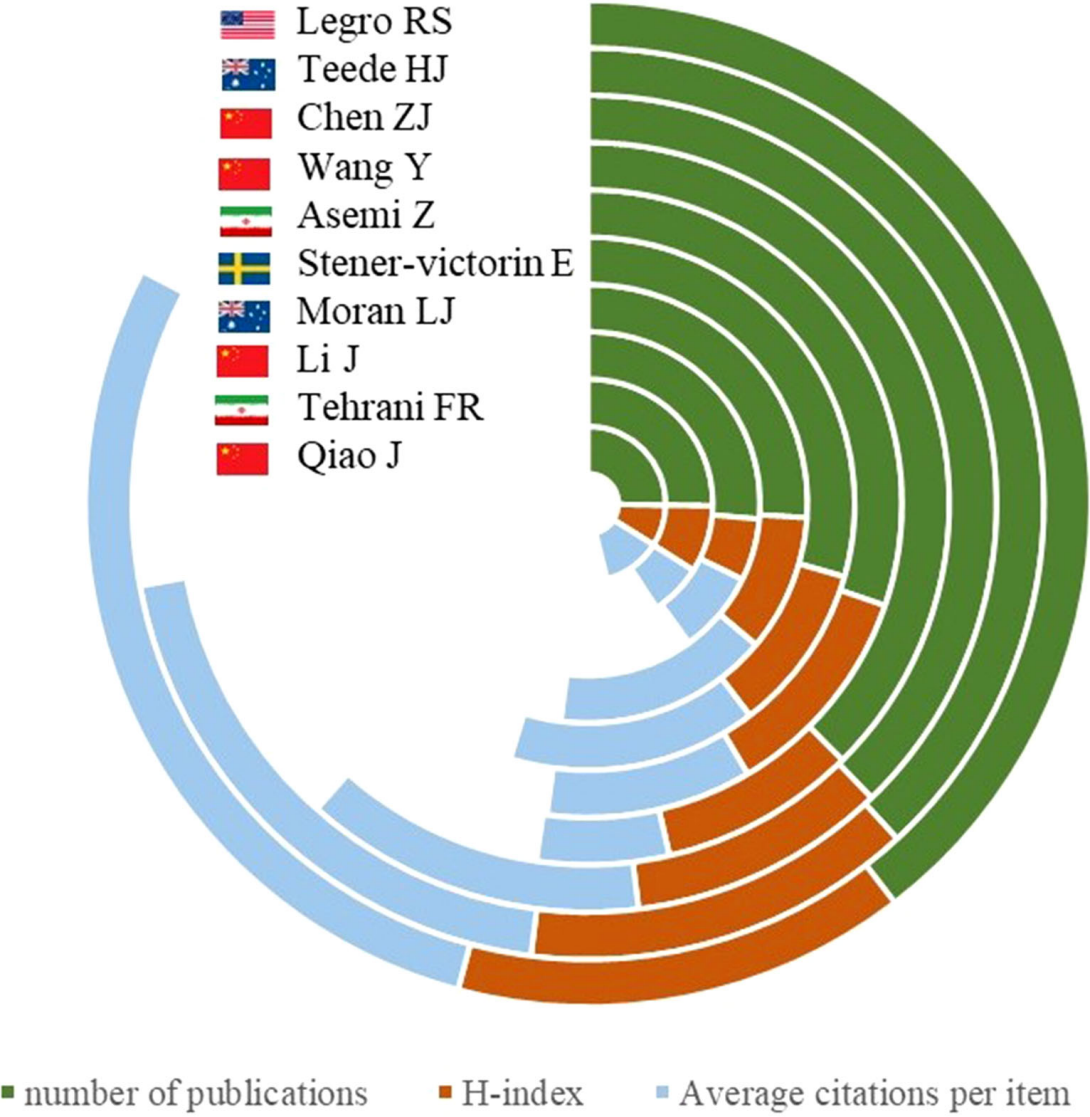
The top 10 authors in terms of total number of publications, ACI, and H-index.

**Figure 6** lists the top 10 most active authors of the PCOS-related publications. Among the list, Prof. Legro RS from the USA was ranked first with 103 articles, followed by Teede HJ from Australia with 99 articles, and Chen ZJ from China with 96 articles. In addition to the number of publications, various indicators, such as H-index and ACI, could help identify the total impact of the author in the form of citations. In terms of H-index, Legro RS (38), Teede HJ (16), Asemi Z (17), and Chen ZJ (18) were listed as the top four highest cited researchers in the field. Legro RS possessed the highest ACI of 74.17 times from ACI, and Teede HJ was the second with the ACI value of 52.19 times, followed by Moran LJ from Australia (40.85 times).

**Figure 7:**
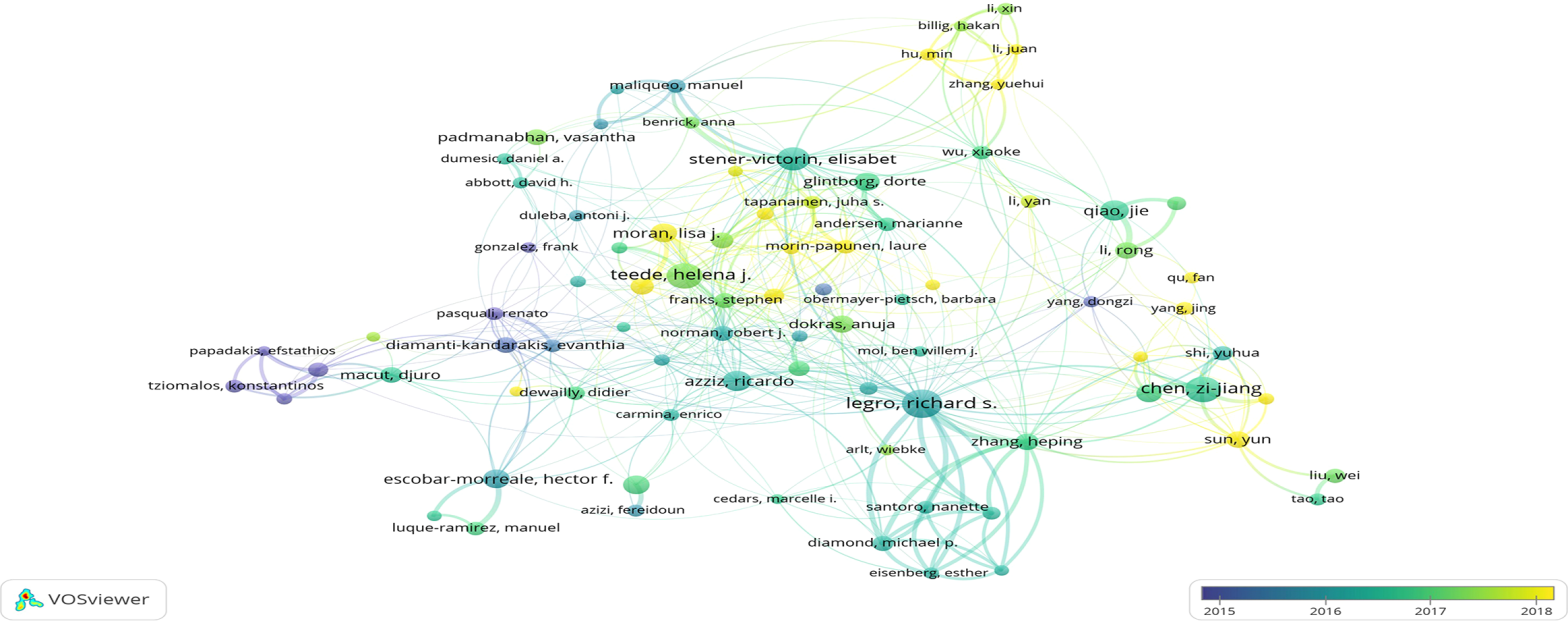
A co-authorship overlay visualization map of authors by VOSviewer.

Further, a co-authorship overlay visualization map of authors with a minimum of 20 articles (**Figure 7**) and a collaboration analysis of core authors in several research clusters (**Supplementary Figure S6**) were presented using VOSviewer. It was observed from these maps that Legro RS, Stener-Victorin E, Chen ZJ, and Teede HJ were positioned in the central location of the co-authorship clusters, but with a few strong links between them, implicating a need to intensify the collaboration and communication among authors in the domain. In addition, the AAY for each author was labeled with different colors, as shown by the gradient at the bottom right. Among these core authors, the contribution of the relatively young researchers should be acknowledged, such as Morin-Papunen L, Moran LJ, Teede HJ, Hu M, and Sun Y, among others.

**Figure 8:**
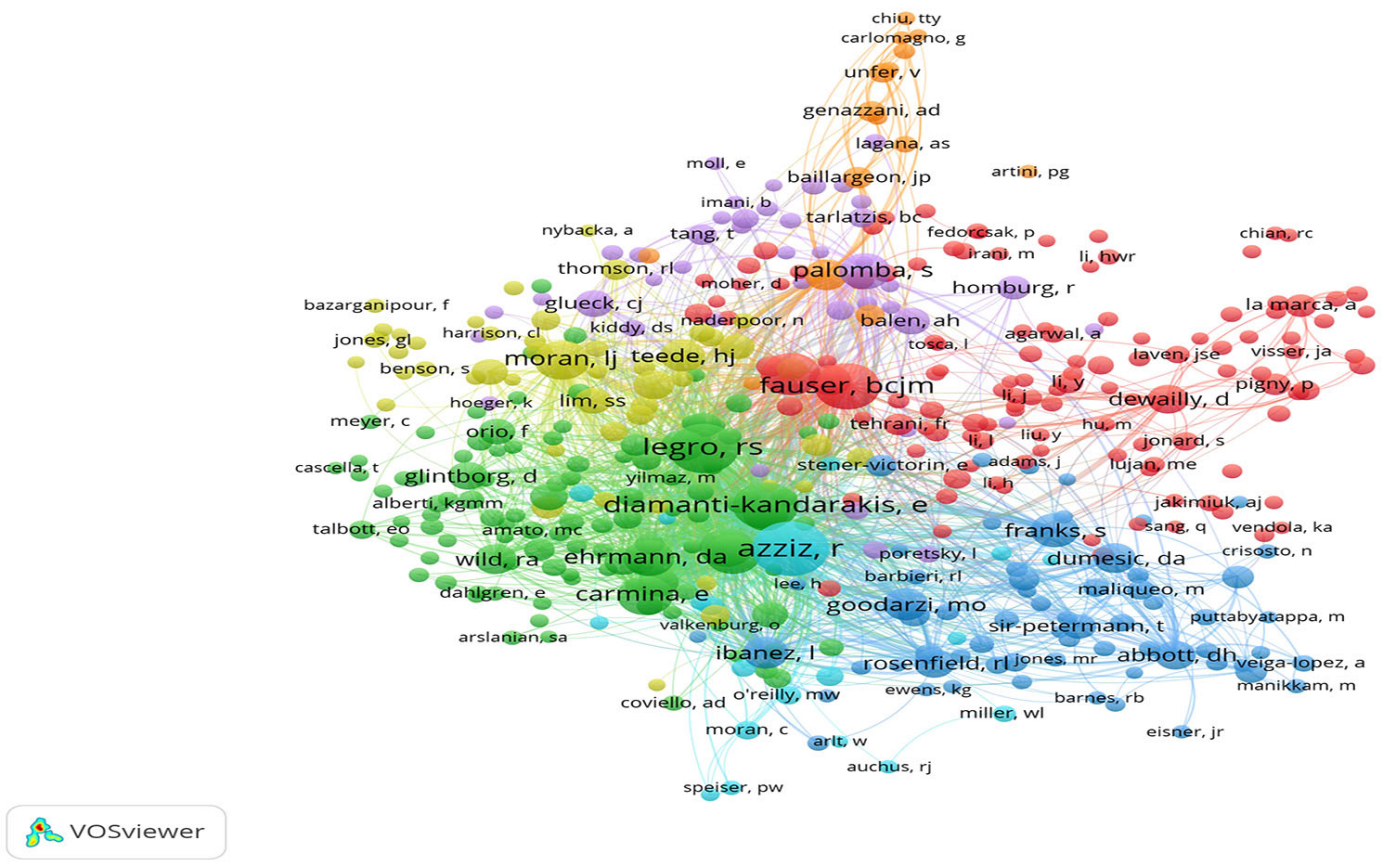
The co-citation network map of authors obtained by the VOSviewer. The node size represents the number of citations. The distance between two nodes indicates the relatedness of authors in terms of co-citation link. The smaller distance, the stronger relatedness, defined as one cluster with the same color.

The co-citation relationship network map of authors with at least 100 citations was obtained by the VOSviewer (**Figure 8**), displaying the authors with a significant influence in this field. The top three authors with the highest TLS included Azziz R, Legro RS, and Diamanti-Kandarakis E. The co-citation relationship of Azziz R with other authors is showed in **Supplementary Figure S7**. Among these authors with more than 100 citations, Azziz R collaborated with 443 authors worldwide.

### Analysis of highly influential journals

The basic information on the top 15 influential journals is summarized in **Table 1**, including citing articles, the journal impact factor (JIF), and H-index. Around 50% of the articles were published in the top 10 most influential journals. Among them, the top three journals with the highest number of citing articles included the *Journal of Clinical Endocrinology & Metabolism*, *Fertility and Sterility*, and *Human Reproduction*. As the JIF and H-index are the essential parameters to measure the quality and value of a journal, *Lancet* possessed the highest JIF of 44.862 and *New England Journal of Medicine* has achieved the second-highest JIF of 19.075.

**Table 1:** Top 15 journals with most publications in PCOS research.

Ranking	Journals title	Citing Articles	JIF (2022)	H-index	Publication country
1	*Journal of Clinical Endocrinology & Metabolism*	8706	5.243	61	United Kingdom
2	*Fertility and Sterility*	7364	3.706	49	Netherlands
3	*Human Reproduction*	7240	5.426	52	United Kingdom
4	*Human Reproduction Update*	6620	12.527	42	United Kingdom
5	*Gynecological Endocrinology*	4480	2.027	33	United Kingdom
6	*Clinical Endocrinology*	4289	2.838	32	United Kingdom
7	*PLoS One*	4064	3.041	36	United States
8	*Endocrine Reviews*	3335	17.27	19	United States
9	*Reproductive Biology and Endocrinology*	3150	4.791	28	United Kingdom
10	*European Journal of Endocrinology*	2692	5.702	30	United Kingdom
11	*Endocrinology*	2276	3.988	34	United Kingdom
12	*New England Journal of Medicine*	1303	19.075	7	United States
13	*Diabetes Care*	937	11.378	11	United States
14	*Lancet*	200	44.862	3	United Kingdom
15	*Diabetes*	682	6.072	9	United States

**Figure 9:**
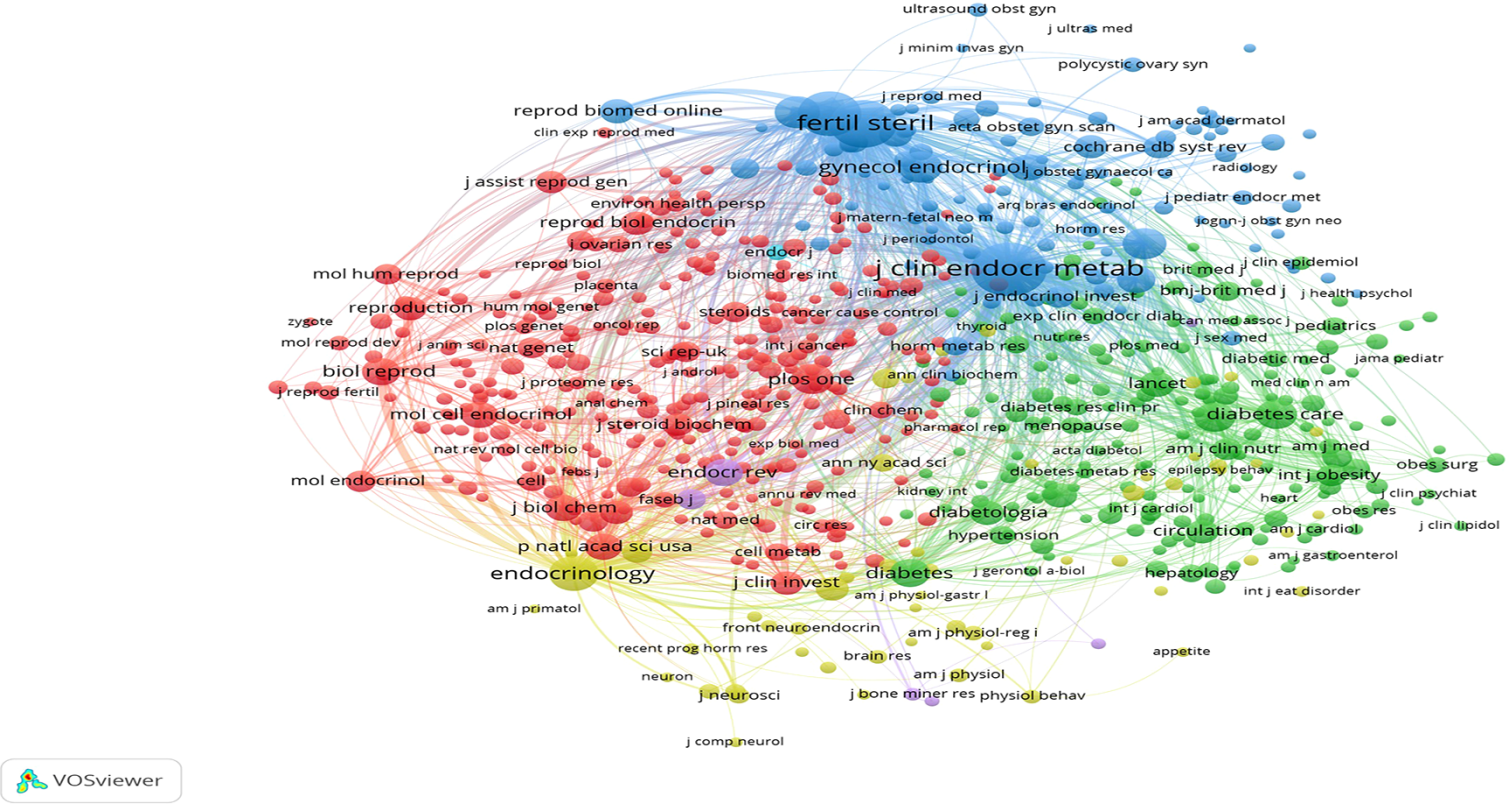
The co-citation network visualization map of journals by VOSviewer.

The co-citation frequency is another valuable parameter to reflect the impact of a journal. The co-citation visualization network map was displayed by the VOSviewer (**Figure 9**), with a minimum of 100 citations of a selected journal. It was observed from the results that the network resulted in 690 nodes and 6 clusters. The top 5 most cited journals included *Journal of Clinical Endocrinology & Metabolism*, *Fertility and Sterility*, *Human Reproduction*, *Endocrinology*, and *Human Reproduction Update*.

### Analysis of the highest relevant subject categories

**Figure 10:**
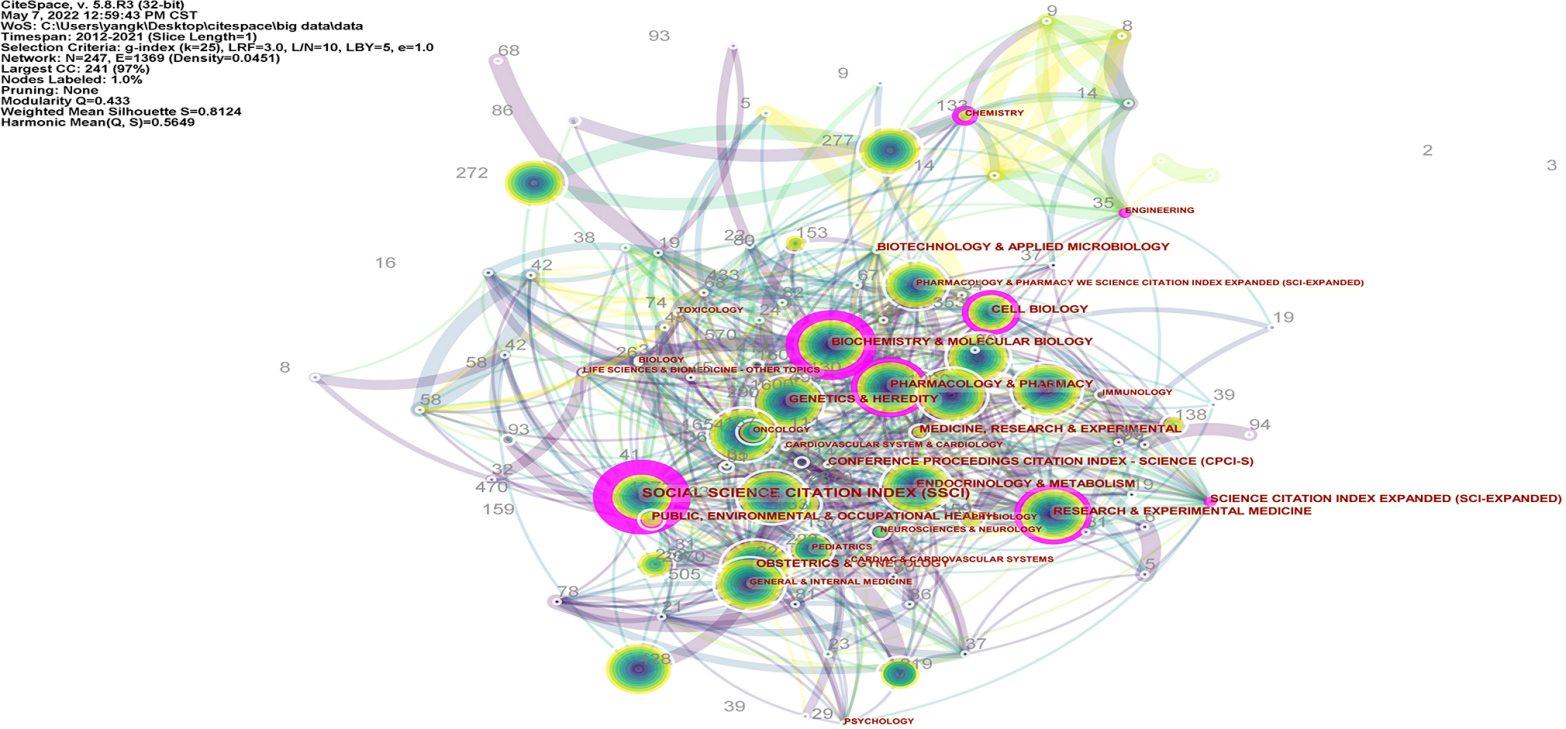
A co-occurring network map of subject categories generated by Citespace.

**Figure 11:**
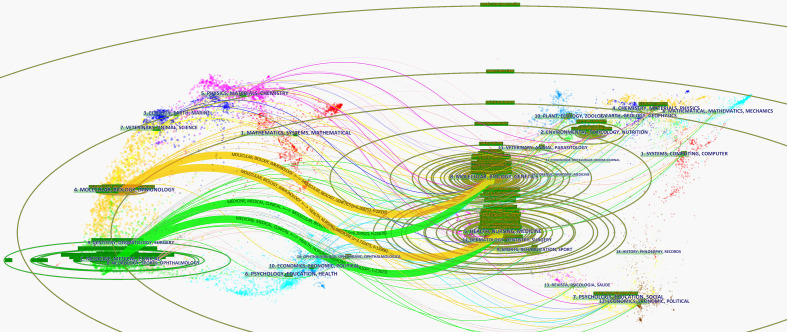
The dual-map overlay of journals stood for the topic distribution of academic journals involving PCOS research (generated by Citespace). The colored connecting lines represent citation relationship. The cited journals were on the right, and the citing journals were on the left.

A co-occurring analysis of subject categories based on journals was generated using CiteSpace (**Figure 10**). The top 15 relevant subject categories with PCOS are displayed in **Supplementary Figure S8**. As can be seen, the three most relevant subject categories correlated with PCOS included Endocrinology & Metabolism, Obstetrics & Gynecology, and Reproductive Biology. Among the relevant subject categories with PCOS, four predominant subject categories with a higher BC value than 0.1, included Biochemistry & Molecular Biology, Pharmacology & Pharmacy, Cell Biology, and Research & Experimental Medicine (from high to low). Otherwise, a dual-map overlay of journals was generated using CiteSpace (**Figure 11**), standing for the citing trajectories for interdisciplinary collaboration. Two primary citation lines tinted in orange were identified. These results indicated that the studies published in Molecular/Biology/Immunology journals mainly cited studies published in the Molecular/Biology/Genetics and Health/Nursing/Medicine journals. In contrast, two main green citation lines showed that a large proportion of articles in Medicine/medical/clinical journals most likely cited articles from the Molecular/Biology/Genetics and Health/Nursing/Medicine journals.

### Analysis of highly cited papers

In this context, the top 20 highest cited papers on PCOS were listed in **Supplementary Table S2**. Among these, the top 5 articles were cited over 900 times. Specifically, a review entitled “Lack of Exercise Is a Major Cause of Chronic Diseases”, published in *Comprehensive Physiology* journal, has been cited 1141 times. The second highest cited paper entitled “Cellular and molecular mechanisms of metformin: an overview”, published in the *Clinical Science*, has been cited 1114 times.

### Co-citation analysis of the references based on citation bursts

Notably, burst detection can significantly classify the cited references that have affected concern among peer researchers. Moreover, the references with citation bursts can assess the developmental frontiers of the domain. The top 25 references with the most robust citation bursts are listed in **Table 2**. The blue line represented the timeline, and the red segment in the blue timeline indicated the duration that a reference showed a burst. Among these top references, the paper with the most substantial citation burst was authored by Azziz R et al. in 2009. The notable second more vigorous burst written by March WA et al.

**Table 2:** The top 30 references co-citation with the strongest citation bursts.

References	Year	Strength	Begin	End	2012 - 2021
Azziz R, 2009, FERTIL STERIL, V91, P456, DOI 10.1016/j.fertnstert.2008.06.035	2009	72.2	2012	2014	▃▃▃▂▂▂▂▂▂▂
March WA, 2010, HUM REPROD, V25, P544, DOI 10.1093/humrep/dep399	2010	53.9	2012	2015	▃▃▃▃▂▂▂▂▂▂
Wild RA, 2010, J CLIN ENDOCR METAB, V95, P2038, DOI 10.1210/jc.2009-2724	2010	53.61	2012	2015	▃▃▃▃▂▂▂▂▂▂
Goodarzi MO, 2011, NAT REV ENDOCRINOL, V7, P219, DOI 10.1038/nrendo.2010.217	2011	41.32	2012	2016	▃▃▃▃▃▂▂▂▂▂
Moran LJ, 2010, HUM REPROD UPDATE, V16, P347, DOI 10.1093/humupd/dmq001	2010	36.07	2012	2015	▃▃▃▃▂▂▂▂▂▂
Chen ZJ, 2011, NAT GENET, V43, P55, DOI 10.1038/ng.732	2011	24.74	2012	2016	▃▃▃▃▃▂▂▂▂▂
Teede H, 2010, BMC MED, V8, P0, DOI 10.1186/1741-7015-8-41	2010	23.64	2012	2015	▃▃▃▃▂▂▂▂▂▂
Palomba S, 2009, ENDOCR REV, V30, P1, DOI 10.1210/er.2008-0030	2009	22.42	2012	2014	▃▃▃▂▂▂▂▂▂▂
Piouka A, 2009, AM J PHYSIOL-ENDOC M, V296, P0, DOI 10.1152/ajpendo.90684.2008	2009	21.32	2012	2014	▃▃▃▂▂▂▂▂▂▂
Fauser BCJM, 2012, FERTIL STERIL, V97, P28, DOI 10.1016/j.fertnstert.2011.09.024	2012	47.38	2013	2017	▂▃▃▃▃▃▂▂▂▂
Diamanti-Kandarakis E, 2012, ENDOCR REV, V33, P981, DOI 10.1210/er.2011-1034	2012	48.25	2014	2017	▂▂▃▃▃▃▂▂▂▂
Shi YY, 2012, NAT GENET, V44, P1020, DOI 10.1038/ng.2384	2012	21.17	2014	2017	▂▂▃▃▃▃▂▂▂▂
Yildiz BO, 2012, HUM REPROD, V27, P3067, DOI 10.1093/humrep/des232	2012	21.03	2014	2017	▂▂▃▃▃▃▂▂▂▂
Stepto NK, 2013, HUM REPROD, V28, P777, DOI 10.1093/humrep/des463	2013	19.17	2014	2018	▂▂▃▃▃▃▃▂▂▂
Lim SS, 2012, HUM REPROD UPDATE, V18, P618, DOI 10.1093/humupd/dms030	2012	19.17	2014	2017	▂▂▃▃▃▃▂▂▂▂
Legro RS, 2013, J CLIN ENDOCR METAB, V98, P4565, DOI 10.1210/jc.2013-2350	2013	59.23	2015	2018	▂▂▂▃▃▃▃▂▂▂
Li R, 2013, HUM REPROD, V28, P2562, DOI 10.1093/humrep/det262	2013	20.53	2015	2018	▂▂▂▃▃▃▃▂▂▂
Conway G, 2014, EUR J ENDOCRINOL, V171, P0, DOI 10.1530/EJE-14-0253	2014	25.5	2016	2019	▂▂▂▂▃▃▃▃▂▂
Sirmans SM, 2014, CLIN EPIDEMIOL, V6, P1, DOI 10.2147/CLEP.S37559	2014	20.64	2016	2019	▂▂▂▂▃▃▃▃▂▂
Dumesic DA, 2015, ENDOCR REV, V36, P487, DOI 10.1210/er.2015-1018	2015	29.67	2017	2021	▂▂▂▂▂▃▃▃▃▃
Goodman NF, 2015, ENDOCR PRACT, V21, P1415, DOI 10.4158/EP15748.DSCPT2	2015	19.19	2017	2021	▂▂▂▂▂▃▃▃▃▃
Bozdag G, 2016, HUM REPROD, V31, P2841, DOI 10.1093/humrep/dew218	2016	41.34	2018	2021	▂▂▂▂▂▂▃▃▃▃
Azziz R, 2016, NAT REV DIS PRIMERS, V2, P0, DOI 10.1038/nrdp.2016.57	2016	39.54	2018	2021	▂▂▂▂▂▂▃▃▃▃
Rosenfield RL, 2016, ENDOCR REV, V37, P467, DOI 10.1210/er.2015-1104	2016	40.79	2019	2021	▂▂▂▂▂▂▂▃▃▃
Teede HJ, 2018, FERTIL STERIL, V110, P364, DOI 10.1016/j.fertnstert.2018.05.004	2018	37.11	2019	2021	▂▂▂▂▂▂▂▃▃▃
Teede HJ, 2018, HUM REPROD, V33, P1602, DOI 10.1093/humrep/dey256	2018	28.37	2019	2021	▂▂▂▂▂▂▂▃▃▃
Teede HJ, 2018, CLIN ENDOCRINOL, V89, P251, DOI 10.1111/cen.13795	2018	34.01	2019	2021	▂▂▂▂▂▂▂▃▃▃
Lizneva D, 2016, FERTIL STERIL, V106, P6, DOI 10.1016/j.fertnstert.2016.05.003	2016	35.11	2019	2021	▂▂▂▂▂▂▂▃▃▃
Tata B, 2018, NAT MED, V24, P834, DOI 10.1038/s41591-018-0035-5	2018	21.66	2019	2021	▂▂▂▂▂▂▂▃▃▃
Day F, 2018, PLOS GENET, V14, P0, DOI 10.1371/journal.pgen.1007813	2018	20.13	2019	2021	▂▂▂▂▂▂▂▃▃▃

### Analysis of co−occurring keywords

**Figure 12:**
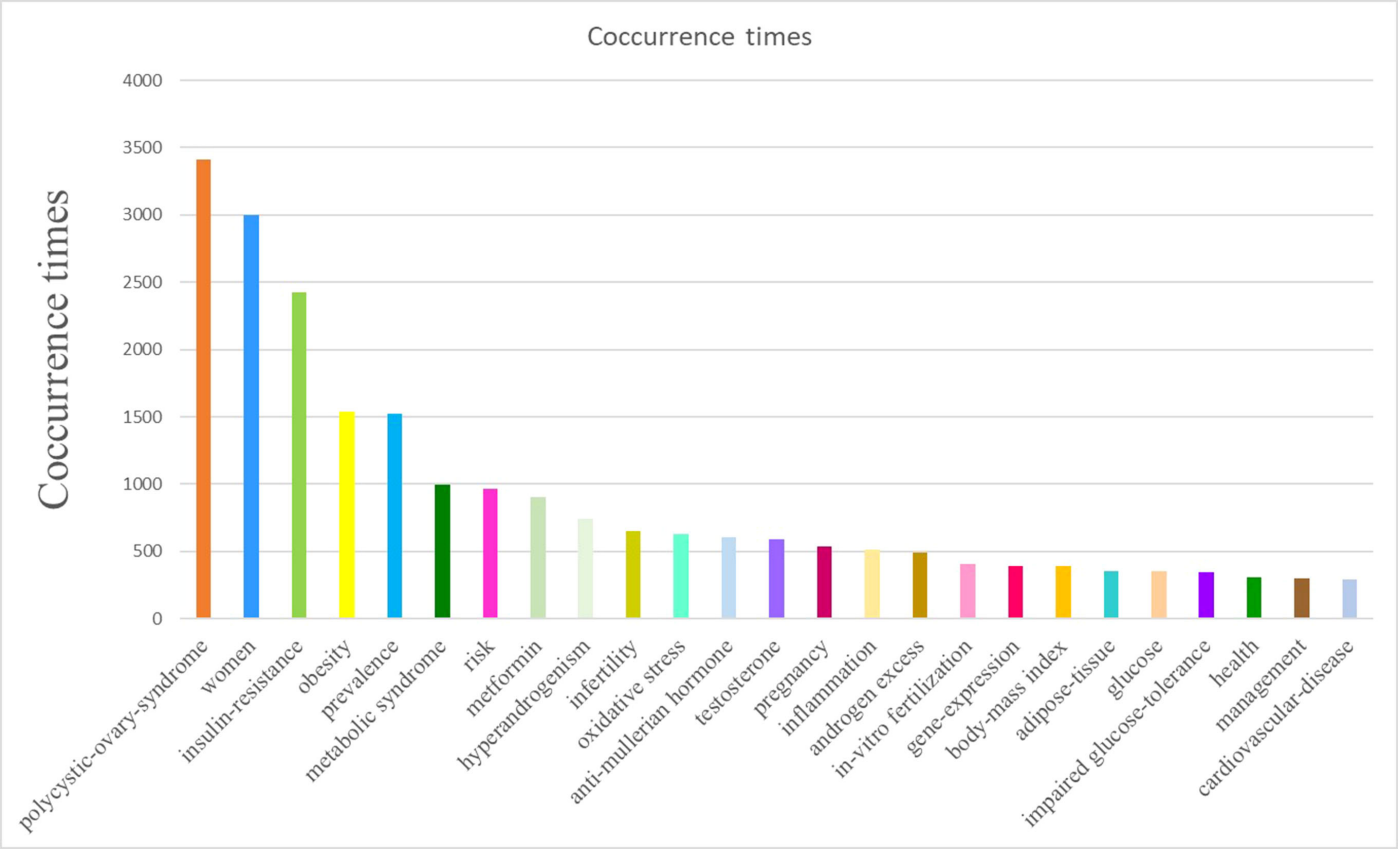
The top 25 keywords with the most frequent occurrences.

The analysis of co-occurring keywords is one of the prevalent ways to identify the main topics of a specific subject area. In this study, a total of 20709 keywords were extracted from 9936 publications. After excluding keywords with no significance and merging keywords with similar meaning, a network visualization map with 518 keywords that occurred at least 30 times was performed using the VOSviewer (**Supplementary Figure S9**). The top 25 most frequently occurred keywords are displayed in **Figure 12**. Apart from “polycystic ovary syndrome” and “women”, the other keywords with frequent occurrences were mainly selected, focusing on pathogenesis (oxidative stress, AMH, gene expression, and inflammation), characteristics (insulin resistance, obesity, and hyperandrogenism), and complication risks (metabolic syndrome, impaired glucose-tolerance, infertility, and cardiovascular diseases).

**Figure 13:**
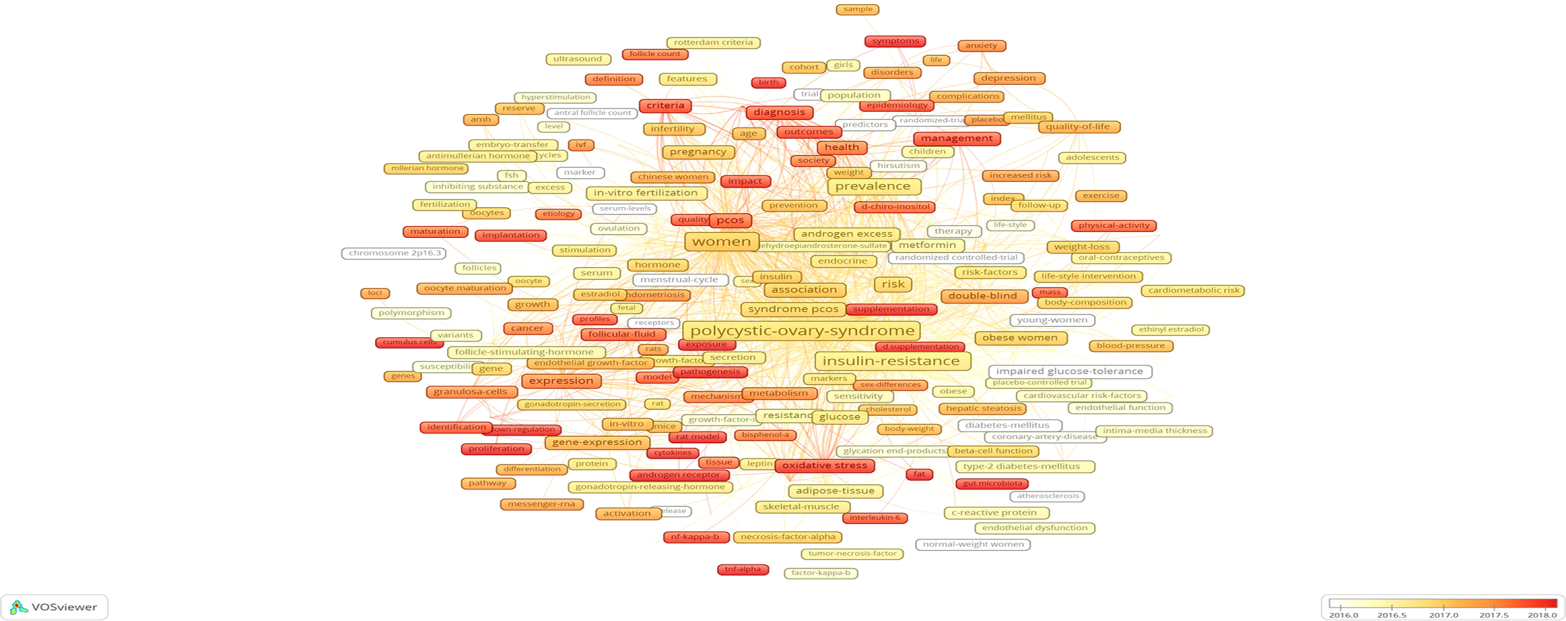
Overlay visualization map of keywords with co-occurrence. The color of each note is based on the average year (AAY) of keyword, as shown by the color gradient at the bottom right.

In addition, the co-occurrence of keywords was colored according to the AAY using VOSviewer (**Figure 13**). In this context, several predominantly used keywords, such as “gut microbiota”, “microRNAs”, “cumulus cells”, “apoptosis”, “Myo-inositol”, “TNF-alpha”, “androgen receptor”, and “Vitamin D-deficient”, showed relatively latest AAY, indicating the potential research topics in the near future.

## Discussion

Based on the analysis of global trends of publications and citations in the field of PCOS, in the past decade (2012-2021), there had been a gradually increasing trend in the annual publications and a rapidly growing trend of citations. The increasing trends indicated that significant attention had been paid to PCOS by researchers towards improved insights in terms of the pathogenesis of PCOS.

Among the top 20 high-yield countries, the USA was the leading country in the academic impact and quality of publications. Although the number of publications from China was highest, the quality was suboptimal, predominantly due to the lack of sufficient citations as one new entrant. Thus, much effort is still needed by China to enhance the quality of the publications and strengthen further collaborations with other countries.

As the most productive institution and with the highest H-index, Monash University consistently brought international leadership in this field. One of his most important achievements was that he spearheaded development and implementation of International evidence-based guideline for the assessment and management of polycystic ovary syndrome 2018. University of Adelaide, although not high in publication counts, ranked first in ACI and second in H-index. Robinson Research Institute at University of Adelaide had a collective of internationally renowned researchers in reproductive health, obstetrics, and gynecology. The likely predominant reason could be that several consensus guidelines from this institution have gained considerable attention. For instance, an article reported the third PCOS consensus workshop on women’s health in 2010, followed by the two previous ESHRE/ASRM-sponsored PCOS consensus. It clarified some knowledge gaps in understanding the women’s health of PCOS and addressed diverse care aspects during the reproductive and post-reproductive years (6). In addition, a systematic review and meta-analysis substantially explored the association between PCOS and obesity, presenting the prevention and management of overweight and obesity in the clinical management of PCOS (17). More often, one of those was considered an international evidence-based guideline regarding some recommendations on assessing and managing PCOS (18).

The co-authorship network analysis mainly provided three significant cooperation network, predominantly concentrated in institutions from Australia, USA, and China, respectively. Of these, Monash University had strongest collaborative ability, followed by the University of Adelaide and the University of Pennsylvania. However, the USA, Australia and China as the leaders in this domain, needed to strengthened further collaboration and communication in different research institutions, specifically with institutions from different countries, to eliminate the academic barriers. One additional tight cluster, formed by many institutions from Iran, was indistinctly placed from the above significant clusters. For instance, a study related to a large-sample epidemiological investigation on PCOS prevalence study based on community from Shahid Beheshti University Medical Sciences has been cited 154 times (19). Notably, another research team from Kashan University of Medical Sciences reported the effect of calcium plus vitamin D supplementation on glucose metabolism and lipid profiles in PCOS for the first time (20), which has been cited 83 times.

Financial support is considered an essential pillar for the developing PCOS research. As can be seen, 80% of the identified articles were supported by these funding agencies in the USA and China. Greater investments was associated with more rapid progress of the field.

Regarding the most prolific authors, Legro RS and Teede HJ were the two most contributed pioneers in this field. In 1999, Legro RS and colleagues published a study about the prevalence and risk of impaired glucose intolerance and type 2 diabetes mellitus in PCOS women. The authors reported that the prevalence of impaired glucose intolerance and type 2 diabetes mellitus were significantly augmented in the PCOS women (21), which has been cited 1285 citations so far. Additionally, Legro RS developed a clinical practice guideline for the diagnosis and treatment of PCOS with additional expert panelists from the Endocrine Society in 2013 (22), which has been cited 878 times. Meanwhile, Teede HJ and group found a high prevalence of IR in PCOS, related to BMI but not visceral fat (23). Alternatively, Teede HJ et al. summarized the guideline recommendations for assessing and managing PCOS (18), providing broader implications in terms of clear advice for clinicians.

The author co-citation relationship refers to the appearance of two authors in the bibliography of a third citing article, revealing the rigidity of the research directions and the impact of the authors (24). As the top-cited author, Prof. Azziz R is an internationally renowned expert in the fields of reproduction and endocrinology, working primarily at the University of Alabama at Birmingham. He was one of the makers of the Rotterdam criteria of PCOS in 2003 and the AES criteria in 2006. Prof. Legro RS, the director of the Department of Obstetrics and Gynecology at Penn State Health, contributions have been mentioned above. Diamanti-Kandarakis E is full professor of the Department of Endocrinology and Diabetes at Hygeia Hospital, Athens, Greece. In 2009, Diamanti-Kandarakis E and Azziz R et al. collaborated to develop the Androgen Excess-PCOS Society (AE-PCOS) criteria, in which hyperandrogenism was the principal aspect in diagnosis in combination with ovarian dysfunction. Further, more emphasis should be given for the contribution of the relatively young researchers.

A comprehensive analysis of journal indicators substantially provides a conducive reference for researchers to quickly find articles or submit manuscripts (25). The top three citing journals in the field of PCOS were the *Journal of Clinical Endocrinology & Metabolism*, *Fertility and Sterility*, and *Human Reproduction*. *Lancet* and *New England Journal of Medicine* were the two journals with the highest JIF. The two journals were the world’s leading medical journals involving all aspects of human health with global coverage in focus. Notably, most publishers were from the UK and the USA except one from the Netherlands. The plausible reason for having no journals from Asian countries might be non-English native language. Nevertheless, China must create some international journals to strengthen its academic impact in the field. Applaudingly, there was immense contribution to the investment in research and development funds from China. Among the top 5 most cited journals, *Fertility and Sterility*, *Human Reproduction*, and *Human Reproduction Update* mainly concerned the field of reproductive medicine. At the same time, *Journal of Clinical Endocrinology & Metabolism* and *Endocrinology* highlighted current topics in endocrinology and metabolism. Notably, the vast amounts of high-quality studies published in these journals have garnered significant attention from researchers working in the area of PCOS.

The number of citations of a paper could directly reflect its influence in the field. The highest cited review, entitled “Lack of Exercise Is a Major Cause of Chronic Diseases”, published in *Comprehensive Physiology* journal, discussed that lack of exercise was the primary cause of most chronic diseases like PCOS, indicating that physical activity could substantially prevent and treat PCOS. The second highest cited paper entitled “Cellular and molecular mechanisms of metformin: an overview”, published in the *Clinical Science*, discussed that metformin could restore ovarian function in PCOS by improving the insulin sensitivity of the ovarian cells and further exploring its underlying molecular mechanisms. The third article, entitled “Diagnosis and Treatment of Polycystic Ovary Syndrome: An Endocrine Society Clinical Practice Guideline”, was illustrated in the discussion in “the most prolific authors” section. The review, entitled “Exercise as medicine - evidence for prescribing exercise as therapy in 26 different chronic diseases”, stated for the importance of exercise as medicine in the treatment of PCOS. In another article, titled “Insulin Resistance and the Polycystic Ovary Syndrome Revisited: An Update on Mechanisms and Implications”, it described the main probable mechanism of insulin resistance in the pathogenesis of PCOS, independent of obesity, and the contribution of androgens to insulin resistance in PCOS.

As the reference with the most robust citation bursts, Azziz R et al. in 2009 (26) indicated an expert consensus about the clinical diagnostic criteria of PCOS, which should be defined by the presence of hyperandrogenism (clinical and/or biochemical), together with ovarian dysfunction (oligo-anovulation and/or polycystic ovaries), and the exclusion of related disorders. As the second more vigorous burst, March WA et al. was a large retrospective cohort study concerning the prevalence of PCOS under conflicting diagnostic criteria (27). In this study, the authors found that the prevalence of PCOS using the NIH criteria (8.7 ± 2.0%) was down to twice that obtained with the Rotterdam (17.8 ± 2.8%) and AES criteria (12.0 ± 2.4%). These references with citation bursts could reflect the development of PCOS research during the period from 2012 to 2021.

The hotspots of PCOS research before 2016 were predominantly concentrated on the continuous improvement of diagnostic criteria, and the exploration of etiology and pathogenesis. Then, it was shifted to the impact caused on health across the lifespan as a complex disease with multiple complications (28). Consequently, the significant attention of researchers was turned to the prevention of PCOS and the monitoring and management of long-term complications.

Meanwhile, some in-depth findings provided new insights into the pathogenesis of PCOS, in the cases of some new risk loci for PCOS were recognized (29). Notably, several references with continuous bursts in recent years indicated that these topics were of paramount concerns in the PCOS research. Among them, the pathogenesis of the disease was still one of the focused areas. For instance, Dumesic DA et al. (1) analyzed the pathophysiology of PCOS in terms of molecular genetics and proposed the significant contribution of epigenetic studies to the development of PCOS. In another instance, Rosenfield RL et al. (30) demonstrated that typical functional ovarian hyperandrogenism (FOH) typically showed a higher prevalence of PCOS than atypical FOH. The most common provocative factors of typical FOH included obesity and insulin resistance. Tata B and colleagues demonstrated the critical role of prenatal exposure to AMH excess and subsequent aberrant signaling from the GnRH receptor in the neuroendocrine abnormalities of PCOS (31). The large-scale genome-related studies after 2018 significantly contributed to further investigating pathological processes in PCOS research (32). In addition, the assessment and management of PCOS have garnered increasing attention, resulting in the introduction of the corresponding guidelines, including the evaluation for the risk of its complications, lifestyle management, emotional well-being, and weight loss (18, 33).

It can be seen from the analysis of co-occurring keywords that the glycolipid metabolism disorder played a crucial role in the pathogenesis and progression of PCOS. Hence, metformin showed promising effects in the its application for PCOS treatment. Considering these attributes, the prevalence and the health management of PCOS have become the research hotspots with global attention.

In addition, keywords with relatively latest AAY showed indicating the potential research topics in the near future. For instance, microRNAs are often referred to as endogenous, small non-coding RNAs, which are differentially expressed in serum, whole blood, adipose tissues, granulosa cells, follicular fluid, and other tissues of PCOS patients (34). Previous reports indicated that the differentially expressed microRNAs played significant roles in the pathogenesis of various diseases, including insulin signaling processes, inflammation-related pathways, cell proliferation, and apoptosis, among others (16, 35, 36). Consequently, we stringently believe that microRNAs are of great significance in investigating the molecular mechanism of PCOS pathogenesis as novel potential biomarkers.

## Limitations

Despite the success in exploring the bibliometric analysis of PCOS research, this study suffers from some limitations. First, the data sources were only downloaded from the WoSCC database, but not other relevant databases, which would undoubtedly miss some related studies. Second, SCIE-based reports were only considered, meaning that the excellent findings written in other languages from the non-English-speaking countries were underestimated, for instance, China, the most productive country. Third, some of the high-quality papers published in recent times might have been missed due to low citation frequency. Therefore, continuous efforts are required to focus on the latest studies from other databases, including the non-English language publications.

## Conclusion

In conclusion, this study has summarized the bibliometric analysis focusing on the current status and global trends in the PCOS research over the past decade (2012-2021). To date, the USA is leading the position in the field of PCOS in terms of the total number of publications and total citation frequency, which could be due to excellent funding sources and adequate facilities. Nevertheless, China will undoubtedly make its contribution to the advancement of this field with adequate funding support. Among various institutions, Monash University was the most prolific institution with the highest H-index value. Notably, the contribution of University of Adelaide should be highly acknowledged. Among the world-renowned researchers, Legro RS and Teede HJ were the most active and influential authors in recent years, while Azziz R was the most contributing pioneer in this field. In these journals analyzed, the *Journal of Clinical Endocrinology & Metabolism* was the most active journal with the highest number of publications and citations in the PCOS research. According to the current analysis, PCOS pathogenesis has become a long-term forefront of research. In recent years, the health management in PCOS prevention and long-term complications have attracted significant attention from researchers. Besides that, the latest research hotspots include “gut microbiota”, “microRNAs”, “apoptosis”, “Myo-inositol”, “TNF-alpha”, “androgen receptor”, and “Vitamin D-deficient”, warranting further focus of this research.

## Data availability statement

The original contributions presented in the study are included in the article/**Supplementary Material**. Further inquiries can be directed to the corresponding author.

## Author contributions

NS designed the study, analyzed the data, and wrote the manuscript. H-BM supervised the study and revised the manuscript. All authors contributed to the article and approved the submitted version.

## Supplementary material

Supplementary Figure 1 Global trends in the number of annual publications and citations on PCOS research from 2012 to 2021.

Supplementary Figure 2 The visualization of cooperative relationships among countries/regions. Thicker lines represent stronger cooperation.

Supplementary Figure 3 Country co-authorship overlay visualization map generated by VOSviewer. The size of each note represents the number of publications. Collaboration between the two countries becomes more close as the line thickens, which is measured by TLS. The color of each note is based on the average appearing year (AAY) of the country, as shown by the gradient at the bottom right.

Supplementary Figure 4 The top 15 most prolific institutions.

Supplementary Figure 5 The top 15 funding agencies for the support of PCOS research.

Supplementary Figure 6 A collaboration analysis of core authors in the several research clusters.

Supplementary Figure 7 The co-citation relationship of Azziz R with other authors.

Supplementary Figure 8 The top 15 most prolific subject categories with BC value.

Supplementary Figure 9 A network visualization map of keywords co-occurrence analysis. The size of the nodes corresponds to the number of occurrences. The distance between two nodes is representative of the relatedness of co-occurrence links.

Supplementary Table 1 Top 20 high-yield countries/regions related to PCOS research. *Rank, based on the number of total publications.*

Supplementary Table 2 The top 20 highest cited papers on PCOS.

## References

[1] Dumesic DA Oberfield SE Stener-Victorin E Marshall JC Laven JS Legro RS. Scientific statement on the diagnostic criteria, epidemiology, pathophysiology, and molecular genetics of polycystic ovary syndrome. Endocr Rev (2015) 36(5):487–525. doi: 10.1210/er.2015-1018 26426951 PMC4591526

[2] Zhang J Sun Z Jiang S Bai X Ma C Peng Q. Probiotic bifidobacterium lactis V9 regulates the secretion of sex hormones in polycystic ovary syndrome patients through the gut-brain axis. mSystems (2019) 4(2):e00017–19. doi: 10.1128/mSystems.00017-19 31020040 PMC6469956

[3] Li R Zhang Q Yang D Li S Lu S Wu X. Prevalence of polycystic ovary syndrome in women in China: a large community-based study. Hum Reprod (2013) 28(9):2562–9. doi: 10.1093/humrep/det262 23814096

[4] Hull MG. Epidemiology of infertility and polycystic ovarian disease: endocrinological and demographic studies. Gynecol Endocrinol (1987) 1(3):235–45. doi: 10.3109/09513598709023610 3140583

[5] Palomba S de Wilde MA Falbo A Koster MP La Sala GB Fauser BC. Pregnancy complications in women with polycystic ovary syndrome. Hum Reprod Update (2015) 21(5):575–92. doi: 10.1093/humupd/dmv029 26117684

[6] Fauser BC Tarlatzis BC Rebar RW Legro RS Balen AH Lobo R. Consensus on women's health aspects of polycystic ovary syndrome (PCOS): the Amsterdam ESHRE/ASRM-sponsored 3rd PCOS consensus workshop group. Fertil Steril (2012) 97(1):28–38 e25. doi: 10.1016/j.fertnstert.2011.09.024 22153789

[7] Barry JA Azizia MM Hardiman PJ. Risk of endometrial, ovarian and breast cancer in women with polycystic ovary syndrome: a systematic review and meta-analysis. Hum Reprod Update (2014) 20(5):748–58. doi: 10.1093/humupd/dmu012 PMC4326303 24688118

[8] Laggari V Diareme S Christogiorgos S Deligeoroglou E Christopoulos P Tsiantis J. Anxiety and depression in adolescents with polycystic ovary syndrome and Mayer-Rokitansky-Kuster-Hauser syndrome. J Psychosom Obstet Gynaecol (2009) 30(2):83–8. doi: 10.1080/01674820802546204 19533486

[9] Cooper ID. Bibliometrics basics. J Med Libr Assoc (2015) 103(4):217–8. doi: 10.3163/1536-5050.103.4.013 PMC4613387 26512226

[10] Shi M Huang W Shu L Hou G Guan Y Song G. Research on polycystic ovary syndrome: a bibliometric analysis from 2009 to 2019. Gynecol Endocrinol (2021) 37(2):121–5. doi: 10.1080/09513590.2020.1807501 32812809

[11] Synnestvedt MB Chen C Holmes JH. CiteSpace II: visualization and knowledge discovery in bibliographic databases. AMIA Annu Symp Proc (2005) 2005:724–8. PMC1560567 16779135

[12] van Eck NJ Waltman L. Software survey: VOSviewer, a computer program for bibliometric mapping. Scientometrics (2010) 84(2):523–38. doi: 10.1007/s11192-009-0146-3 PMC2883932 20585380

[13] Chen C Dubin R Kim MC. Emerging trends and new developments in regenerative medicine: a scientometric update (2000 - 2014). Expert Opin Biol Ther (2014) 14(9):1295–317. doi: 10.1517/14712598.2014.920813 25077605

[14] Chen C Leydesdorff L. Patterns of connections and movements in dual-map overlays: A new method of publication portfolio analysis. J Assoc Inf Sci Technol (2013) 65(2):334–51. doi: 10.1002/asi.22968

[15] Wang K Xing D Dong S Lin J. The global state of research in nonsurgical treatment of knee osteoarthritis: a bibliometric and visualized study. BMC Musculoskelet Disord (2019) 20(1):407. doi: 10.1186/s12891-019-2804-9 31484517 PMC6727547

[16] Deswal R Dang AS. Dissecting the role of micro-RNAs as a diagnostic marker for polycystic ovary syndrome: a systematic review and meta-analysis. Fertil Steril (2020) 113(3):661–9 e2. doi: 10.1016/j.fertnstert.2019.11.001 32192599

[17] Lim SS Davies MJ Norman RJ Moran LJ. Overweight, obesity and central obesity in women with polycystic ovary syndrome: a systematic review and meta-analysis. Hum Reprod Update (2012) 18(6):618–37. doi: 10.1093/humupd/dms030 22767467

[18] Teede HJ Misso ML Costello MF Dokras A Laven J Moran L. Recommendations from the international evidence-based guideline for the assessment and management of polycystic ovary syndrome. Fertil Steril (2018) 110(3):364–79. doi: 10.1016/j.fertnstert.2018.05.004 PMC6939856 30033227

[19] Tehrani FR Simbar M Tohidi M Hosseinpanah F Azizi F. The prevalence of polycystic ovary syndrome in a community sample of Iranian population: Iranian PCOS prevalence study. Reprod Biol Endocrinol (2011) 9:39. doi: 10.1186/1477-7827-9-39 21435276 PMC3070632

[20] Asemi Z Foroozanfard F Hashemi T Bahmani F Jamilian M Esmaillzadeh A. Calcium plus vitamin d supplementation affects glucose metabolism and lipid concentrations in overweight and obese vitamin d deficient women with polycystic ovary syndrome. Clin Nutr (2015) 34(4):586–92. doi: 10.1016/j.clnu.2014.09.015 25300649

[21] Legro RS Kunselman AR Dodson WC Dunaif A. Prevalence and predictors of risk for type 2 diabetes mellitus and impaired glucose tolerance in polycystic ovary syndrome: a prospective, controlled study in 254 affected women. J Clin Endocrinol Metab (1999) 84(1):165–9. doi: 10.1210/jcem.84.1.5393 9920077

[22] Legro RS Arslanian SA Ehrmann DA Hoeger KM Murad MH Pasquali R. Diagnosis and treatment of polycystic ovary syndrome: an endocrine society clinical practice guideline. J Clin Endocrinol Metab (2013) 98(12):4565–92. doi: 10.1210/jc.2013-2350 PMC5399492 24151290

[23] Stepto NK Cassar S Joham AE Hutchison SK Harrison CL Goldstein RF. Women with polycystic ovary syndrome have intrinsic insulin resistance on euglycaemic-hyperinsulaemic clamp. Hum Reprod (2013) 28(3):777–84. doi: 10.1093/humrep/des463 23315061

[24] Chen Q Fan G Na W Liu J Cui J Li H. Past, present, and future of groundwater remediation research: A scientometric analysis. Int J Environ Res Public Health (2019) 16(20):3975. doi: 10.3390/ijerph16203975 31635235 PMC6843360

[25] Wang C Jing H Sun Z Yao J Zhang X Liu T. A bibliometric analysis of primary aldosteronism research from 2000 to 2020. Front Endocrinol (Lausanne) (2021) 12:665912. doi: 10.3389/fendo.2021.665912 33986730 PMC8111213

[26] Azziz R Carmina E Dewailly D Diamanti-Kandarakis E Escobar-Morreale HF Futterweit W. The androgen excess and PCOS society criteria for the polycystic ovary syndrome: the complete task force report. Fertil Steril (2009) 91(2):456–88. doi: 10.1016/j.fertnstert.2008.06.035 18950759

[27] March WA Moore VM Willson KJ Phillips DI Norman RJ Davies MJ. The prevalence of polycystic ovary syndrome in a community sample assessed under contrasting diagnostic criteria. Hum Reprod (2010) 25(2):544–51. doi: 10.1093/humrep/dep399 19910321

[28] Teede H Deeks A Moran L. Polycystic ovary syndrome: a complex condition with psychological, reproductive and metabolic manifestations that impacts on health across the lifespan. BMC Med (2010) 8:41. doi: 10.1186/1741-7015-8-41 20591140 PMC2909929

[29] Chen ZJ Zhao H He L Shi Y Qin Y Shi Y. Genome-wide association study identifies susceptibility loci for polycystic ovary syndrome on chromosome 2p16.3, 2p21 and 9q33.3. Nat Genet (2011) 43(1):55–9. doi: 10.1038/ng.732 21151128

[30] Rosenfield RL Ehrmann DA. The pathogenesis of polycystic ovary syndrome (PCOS): The hypothesis of PCOS as functional ovarian hyperandrogenism revisited. Endocr Rev (2016) 37(5):467–520. doi: 10.1210/er.2015-1104 27459230 PMC5045492

[31] Tata B Mimouni NEH Barbotin AL Malone SA Loyens A Pigny P. Elevated prenatal anti-mullerian hormone reprograms the fetus and induces polycystic ovary syndrome in adulthood. Nat Med (2018) 24(6):834–46. doi: 10.1038/s41591-018-0035-5 PMC6098696 29760445

[32] Day F Karaderi T Jones MR Meun C He C Drong A. Large-Scale genome-wide meta-analysis of polycystic ovary syndrome suggests shared genetic architecture for different diagnosis criteria. PloS Genet (2018) 14(12):e1007813. doi: 10.1371/journal.pgen.1007813 30566500 PMC6300389

[33] Goodman NF Cobin RH Futterweit W Glueck JS Legro RS Carmina E. American Association of clinical endocrinologists, American college of endocrinology, and androgen excess and pcos society disease state clinical review: Guide to the best practices in the evaluation and treatment of polycystic ovary syndrome - part 2. Endocr Pract (2015) 21(12):1415–26. doi: 10.4158/EP15748.DSCPT2 26642102

[34] Mu L Sun X Tu M Zhang D. Non-coding RNAs in polycystic ovary syndrome: a systematic review and meta-analysis. Reprod Biol Endocrinol (2021) 19(1):10. doi: 10.1186/s12958-020-00687-9 33446212 PMC7807442

[35] Luo Y Cui C Han X Wang Q Zhang C. The role of miRNAs in polycystic ovary syndrome with insulin resistance. J Assist Reprod Genet (2021) 38(2):289–304. doi: 10.1007/s10815-020-02019-7 33405004 PMC7884539

[36] Qin Y Wang Y Zhao H Yang Z Kang Y. Aberrant miRNA-mRNA regulatory network in polycystic ovary syndrome is associated with markers of insulin sensitivity and inflammation. Ann Transl Med (2021) 9(18):1405. doi: 10.21037/atm-21-1288 34733957 PMC8506717

