# Supplementary figures and images for: Global trends in polycystic ovary syndrome research: A 10-year bibliometric analysis
Source: Front Endocrinol (Lausanne). 2023 Jan 9;13:1027945. doi: 10.3389/fendo.2022.1027945 (PMC9868474; doi:10.3389/fendo.2022.1027945)

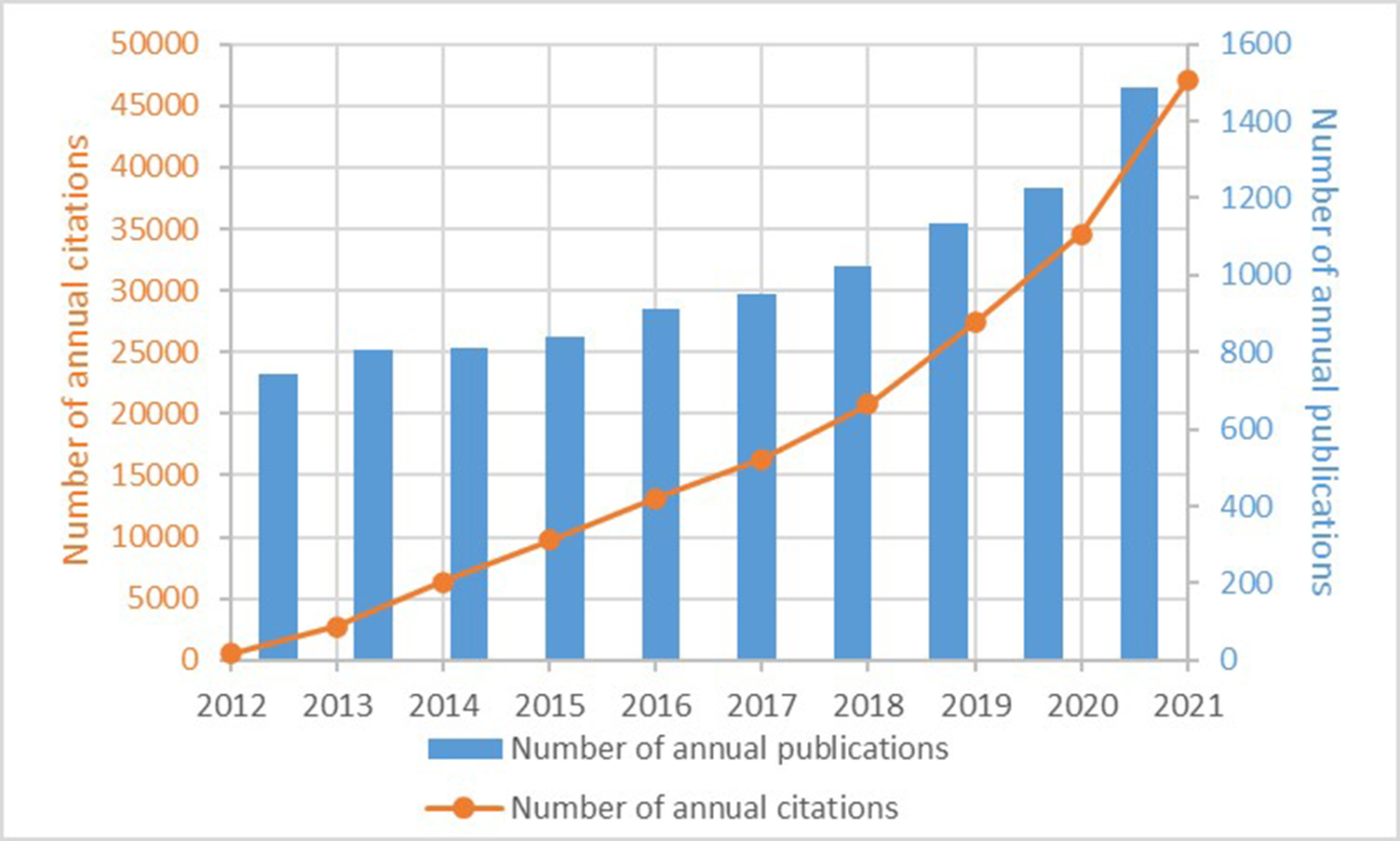

Supplement: Supplementary Figure 1 — Global trends in the number of annual publications and citations on PCOS research from 2012 to 2021. [file Image_1.jpeg]

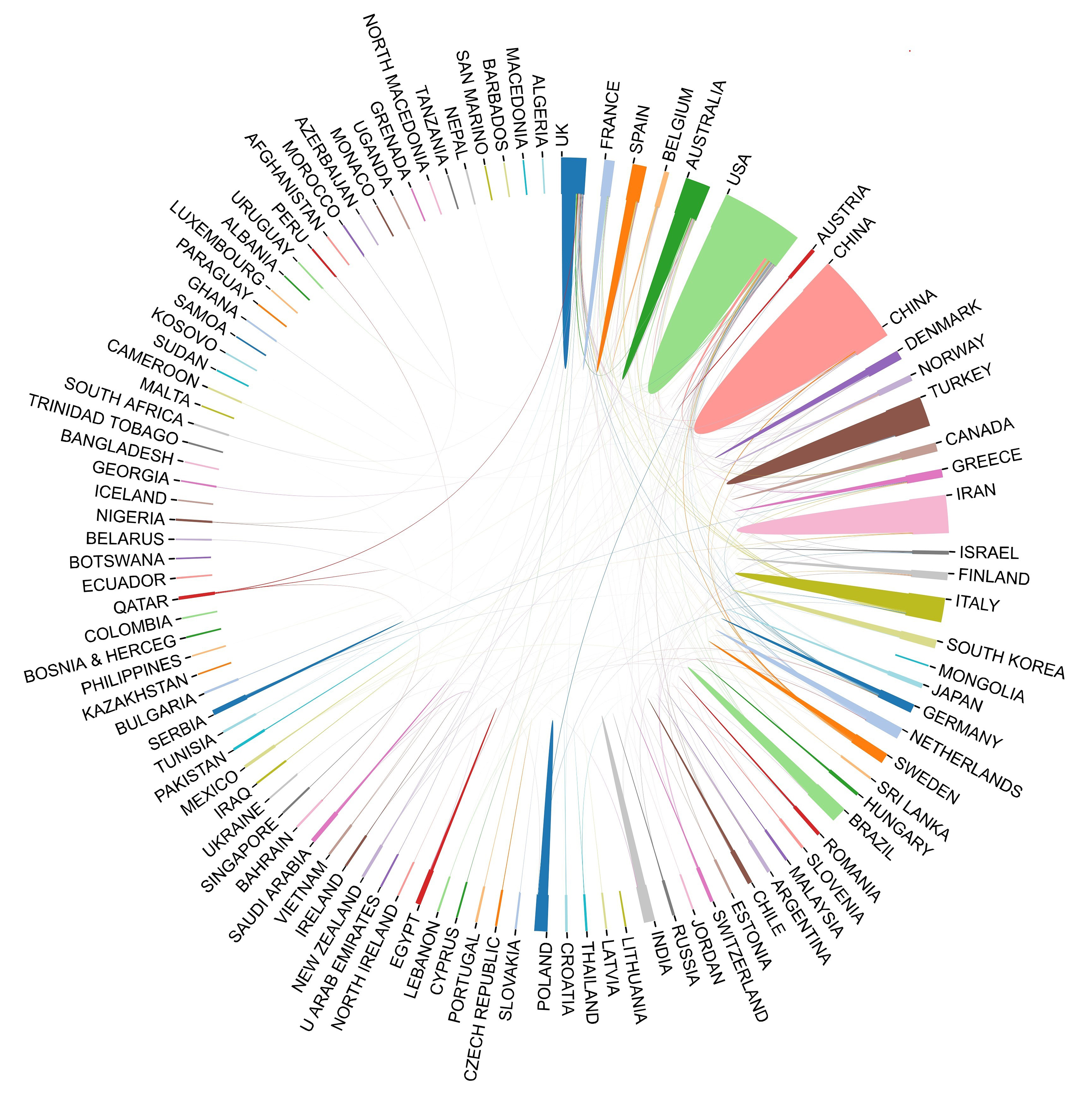

Supplement: Supplementary Figure 2 — The visualization of cooperative relationships among countries/regions. Thicker lines represent stronger cooperation. [file Image_2.jpeg]

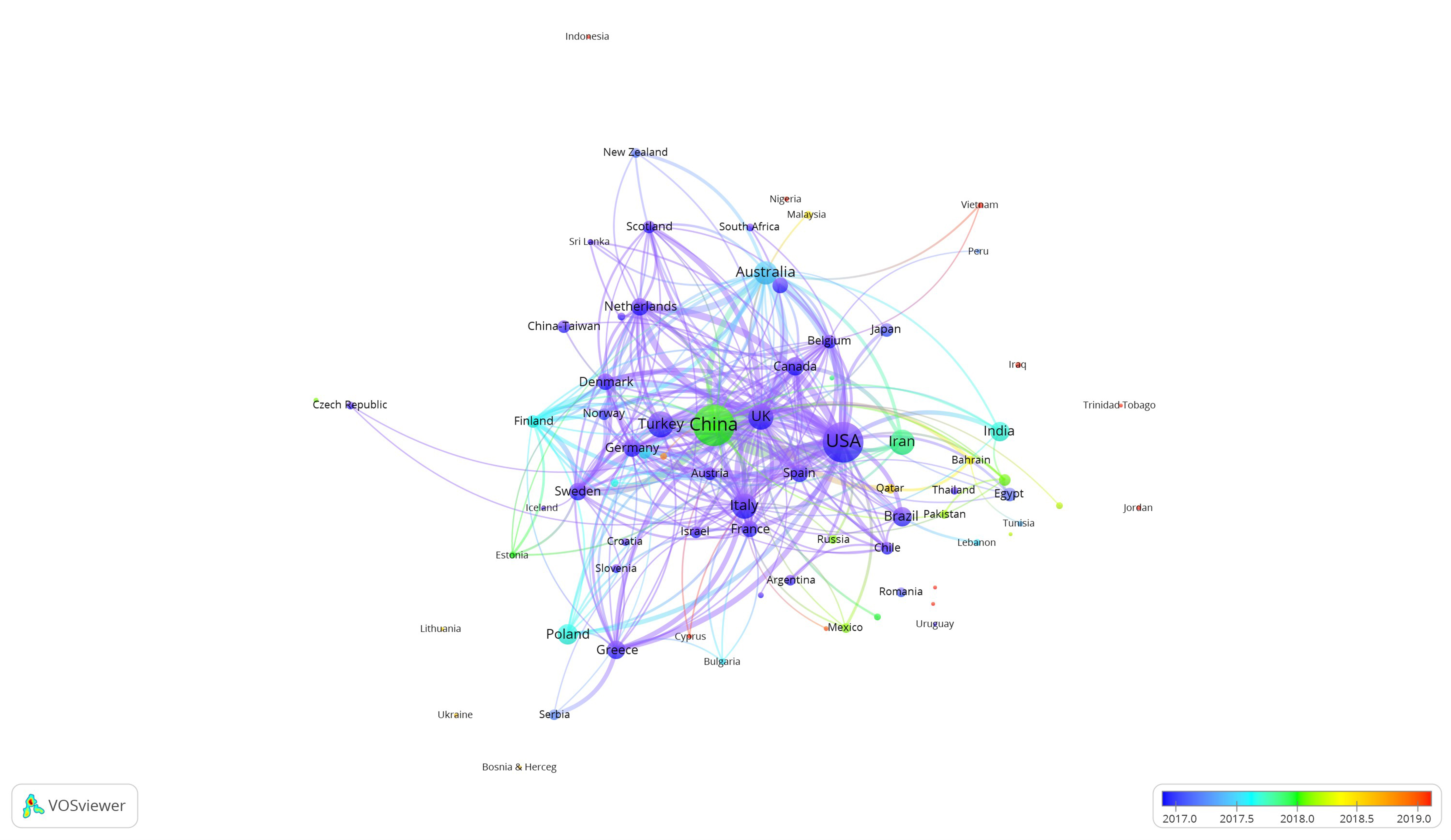

Supplement: Supplementary Figure 3 — Country co-authorship overlay visualization map generated by VOSviewer. The size of each note represents the number of publications. Collaboration between the two countries becomes more close as the line thickens, which is measured by TLS. The color of each note is based on the average appearing year (AAY) of the country, as shown by the gradient at the bottom right. [file Image_3.jpeg]

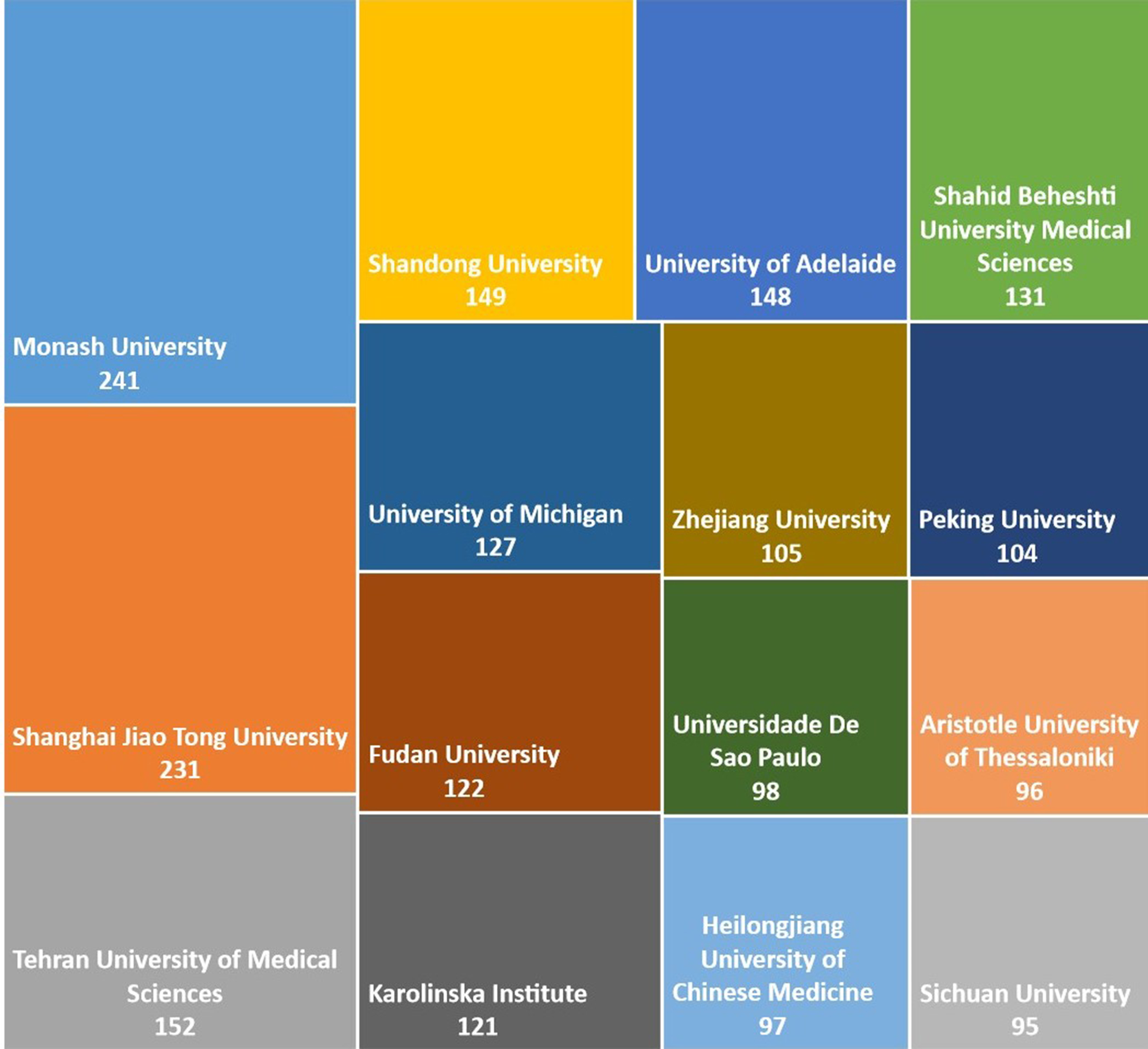

Supplement: Supplementary Figure 4 — The top 15 most prolific institutions. [file Image_4.jpeg]

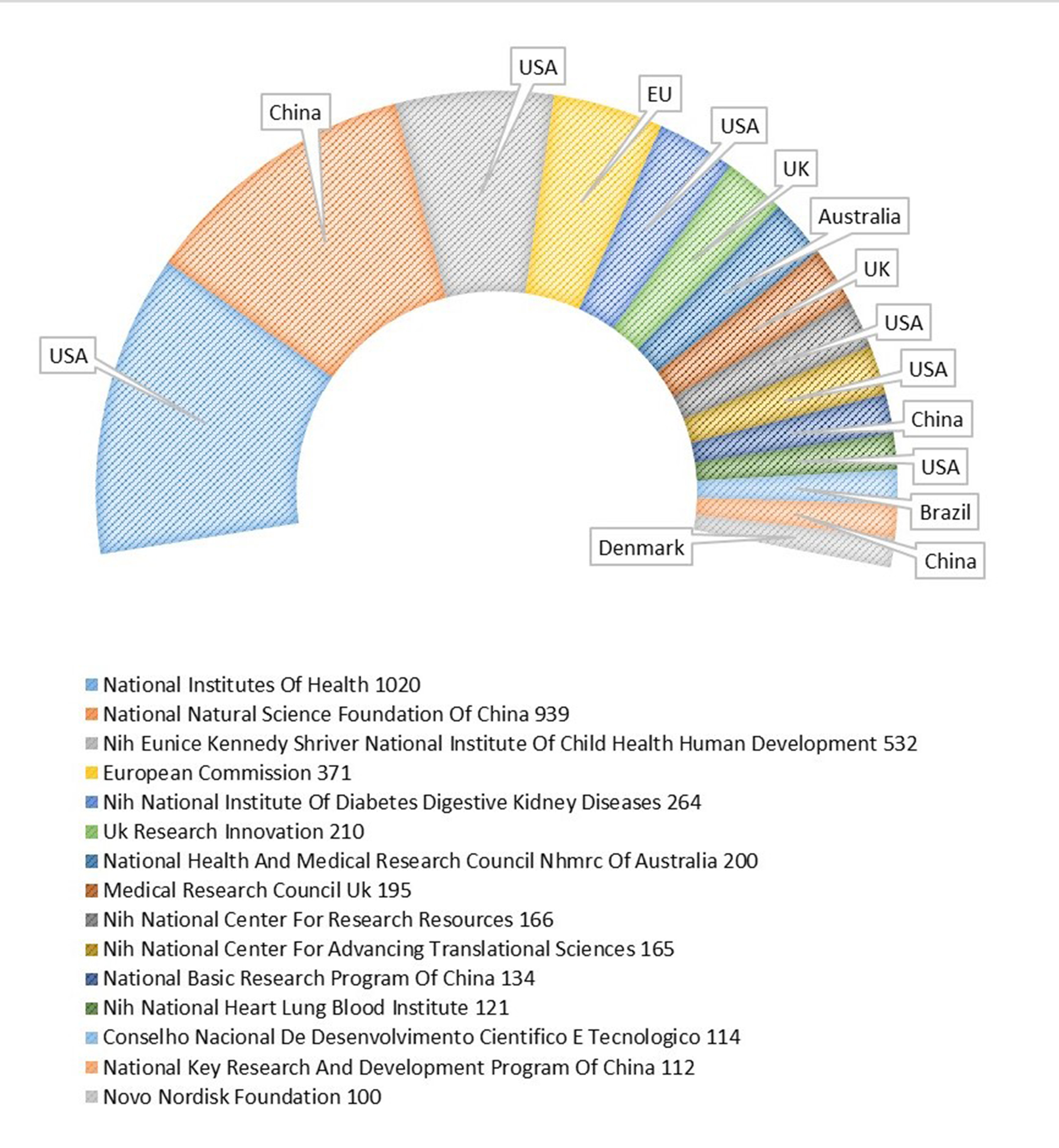

Supplement: Supplementary Figure 5 — The top 15 funding agencies for the support of PCOS research. [file Image_5.jpg]

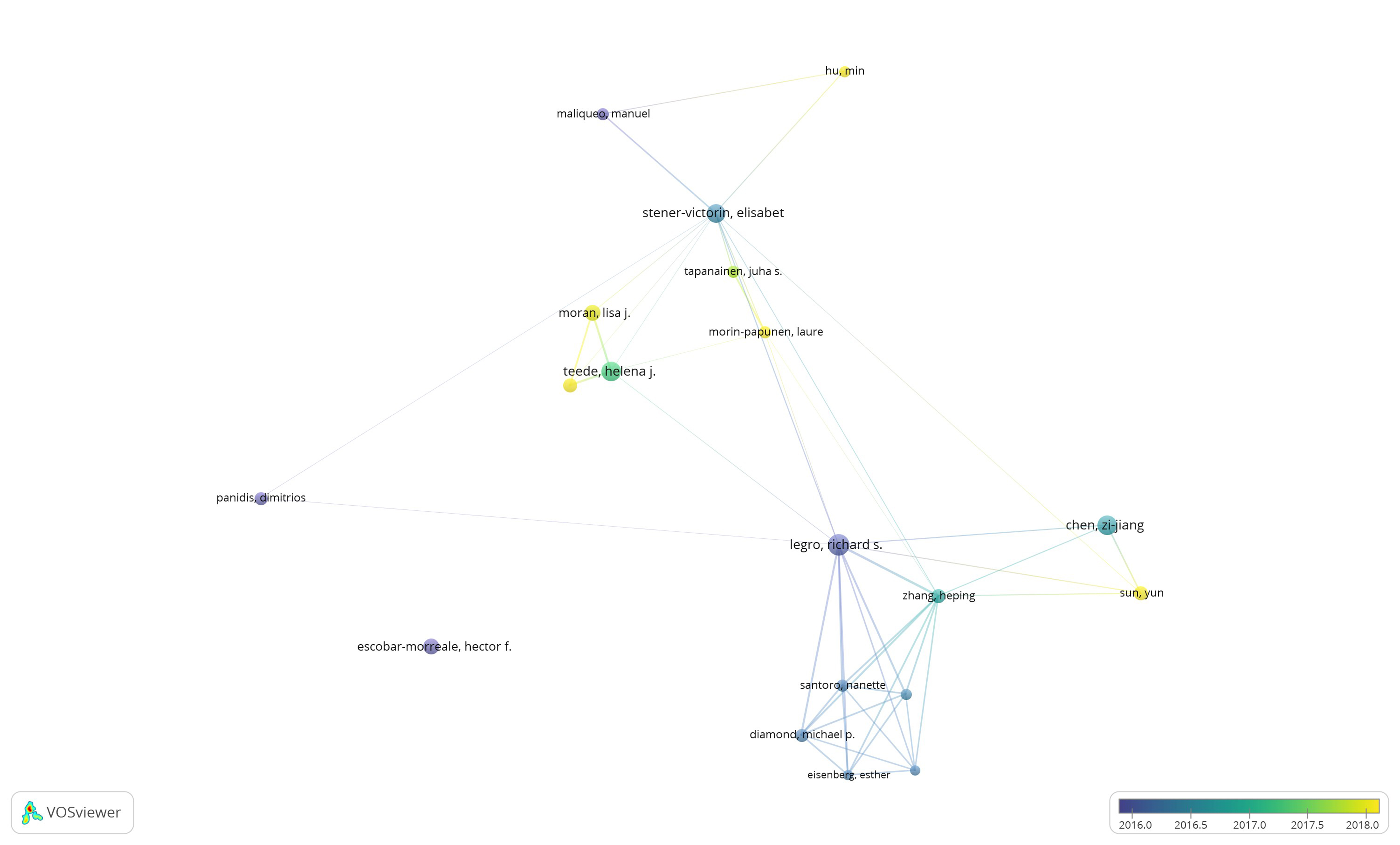

Supplement: Supplementary Figure 6 — A collaboration analysis of core authors in the several research clusters. [file Image_6.jpg]

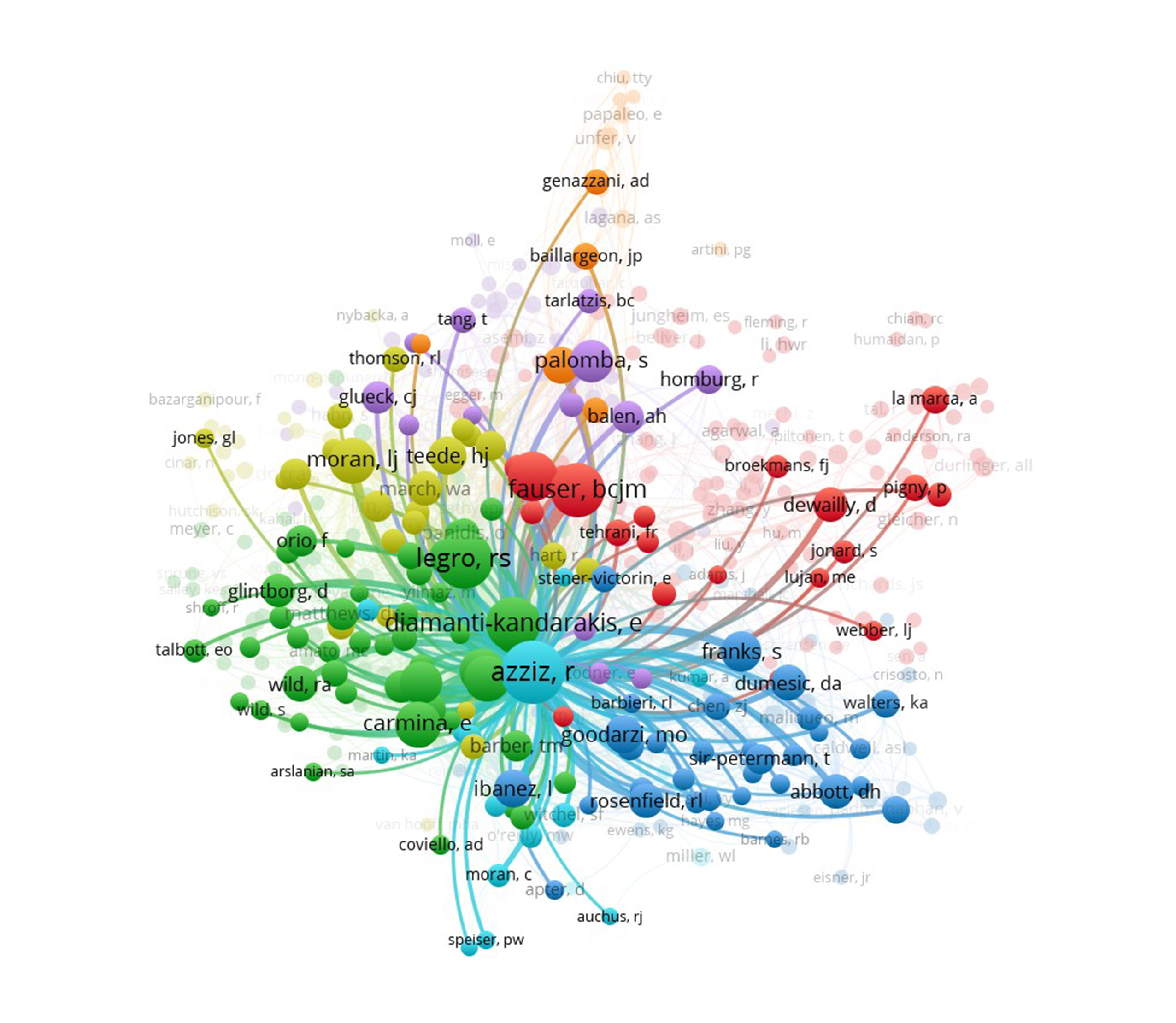

Supplement: Supplementary Figure 7 — The co-citation relationship of Azziz R with other authors. [file Image_7.jpg]

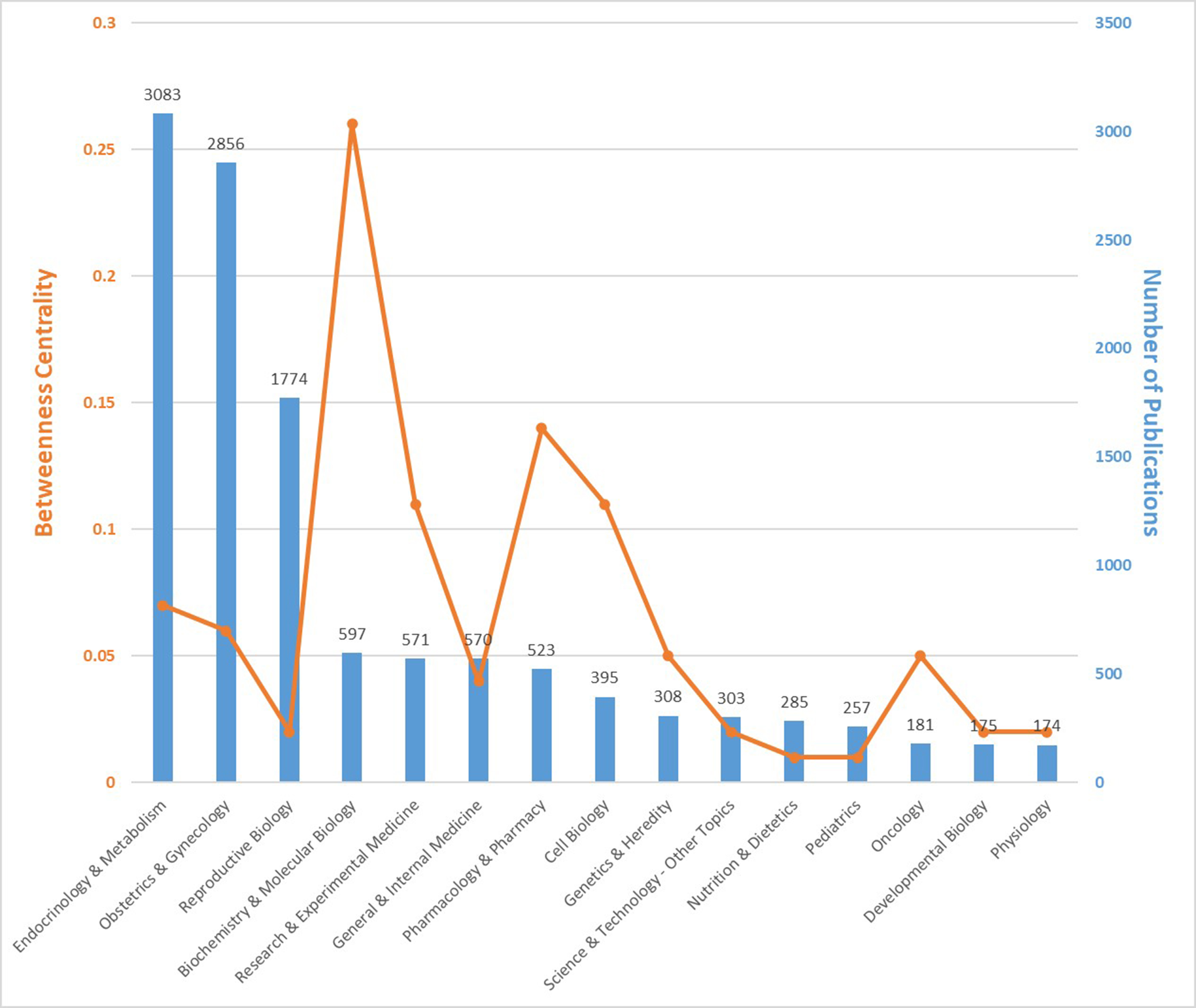

Supplement: Supplementary Figure 8 — The top 15 most prolific subject categories with BC value. [file Image_8.jpg]

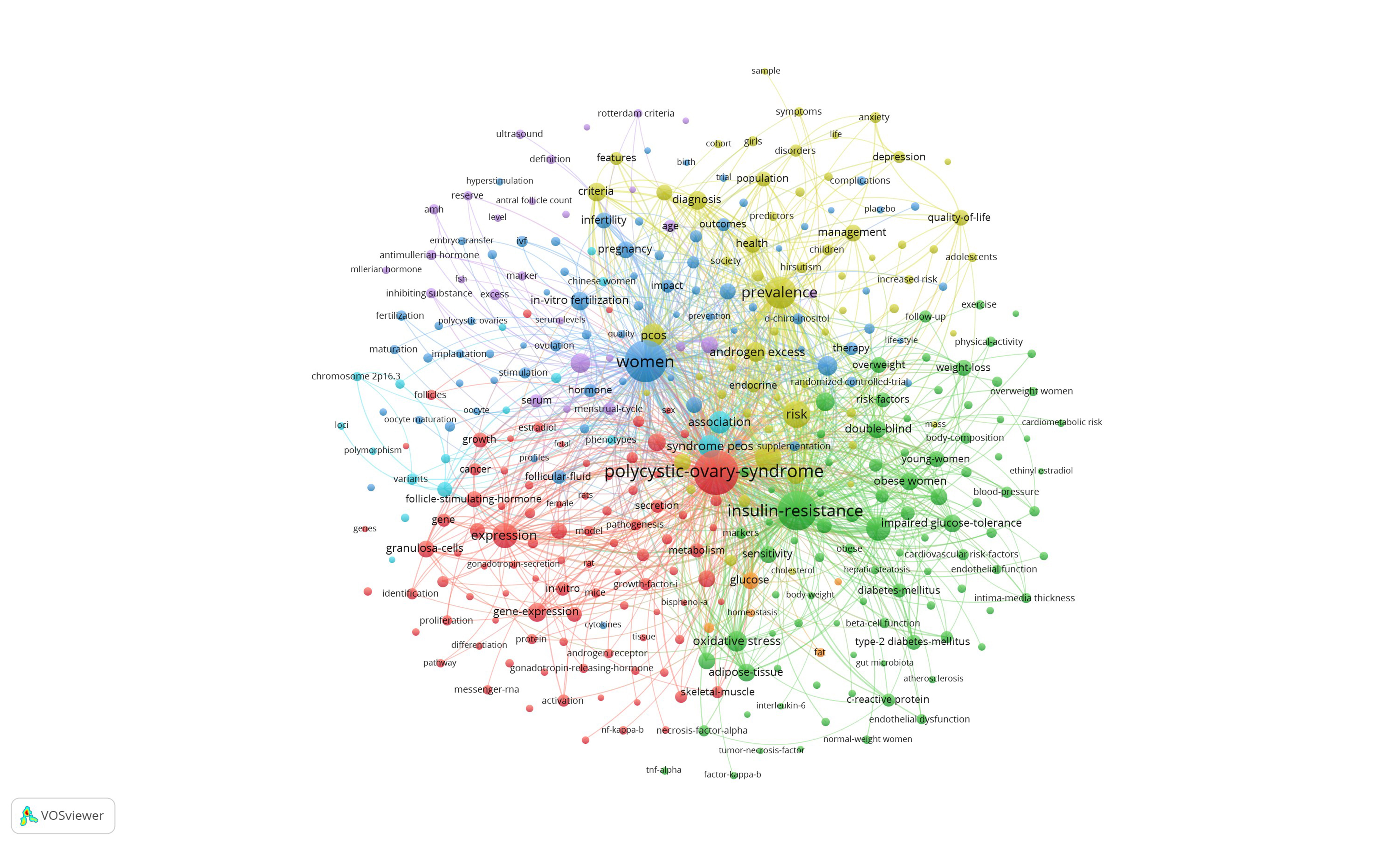

Supplement: Supplementary Figure 9 — A network visualization map of keywords co-occurrence analysis. The size of the nodes corresponds to the number of occurrences. The distance between two nodes is representative of the relatedness of co-occurrence links. [file Image_9.jpg]
