# Supplementary material for: Global trends in polycystic ovary syndrome research: A 10-year bibliometric analysis
Source: Front Endocrinol (Lausanne). 2023 Jan 9;13:1027945. doi: 10.3389/fendo.2022.1027945 (PMC9868474; doi:10.3389/fendo.2022.1027945)
Supplement: Supplementary Table 1 — Top 20 high-yield countries/regions related to PCOS research. Rank, based on the number of total publications. [file Table_1.docx]

**SUPPLEMENTARY TABLE S1** Top 20 high-yield countries/regions related to PCOS research.

| **Rank** | **Country/Region** | **Number of total publications** | **H-index** |
| --- | --- | --- | --- |
| 1 | China | 2154 | 63 |
| 2 | USA | 2101 | 114 |
| 3 | Turkey | 672 | 38 |
| 4 | Italy | 632 | 66 |
| 5 | United Kingdom | 636 | 72 |
| 6 | Iran, Islamic Rep. | 598 | 42 |
| 7 | Australia | 534 | 69 |
| 8 | Poland | 351 | 34 |
| 9 | India | 373 | 38 |
| 10 | Brazil | 317 | 33 |
| 11 | Canada | 291 | 46 |
| 12 | Greece | 262 | 45 |
| 13 | Germany | 252 | 37 |
| 14 | Spain | 238 | 44 |
| 15 | Netherlands | 231 | 54 |
| 16 | Sweden | 228 | 45 |
| 17 | Denmark | 201 | 29 |
| 18 | France | 203 | 50 |
| 19 | Korea, Rep. | 174 | 25 |
| 20 | Egypt, Arab Rep. | 143 | 19 |

*Rank, based on the number of total publications.*
