# Supplementary material for: Global trends in polycystic ovary syndrome research: A 10-year bibliometric analysis
Source: Front Endocrinol (Lausanne). 2023 Jan 9;13:1027945. doi: 10.3389/fendo.2022.1027945 (PMC9868474; doi:10.3389/fendo.2022.1027945)
Supplement: Supplementary Table 2 — The top 20 highest cited papers on PCOS. [file Table_2.docx]

**SUPPLEMENTARY TABLE S2** The top 20 highest cited papers on PCOS.

| **Ranking** | **Title** | **First Author** | **Source Title** | **Publication Year** | **Total Citations** |
| --- | --- | --- | --- | --- | --- |
| 1 | Lack of Exercise Is a Major Cause of Chronic Diseases | Booth FW | *Comprehensive Physiology* | 2012 | 1141 |
| 2 | Cellular and molecular mechanisms of metformin: an overview | Viollet B | *Clinical Science* | 2012 | 1114 |
| 3 | Diagnosis and Treatment of Polycystic Ovary Syndrome: An Endocrine Society Clinical Practice Guideline | Legro RS | *Journal Of Clinical Endocrinology & Metabolism* | 2013 | 927 |
| 4 | Exercise as medicine - evidence for prescribing exercise as therapy in 26 different chronic diseases | Pedersen BK | *Scandinavian Journal of Medicine & Science in Sports* | 2015 | 908 |
| 5 | Insulin Resistance and the Polycystic Ovary Syndrome Revisited: An Update on Mechanisms and Implications | Evanthia Diamanti-Kandarakis | *Endocrine Reviews* | 2012 | 903 |
| 6 | Consensus on women's health aspects of polycystic ovary syndrome (PCOS): the Amsterdam ESHRE/ASRM-Sponsored 3rd PCOS Consensus Workshop Group | Bart C J M Fauser | *Fertility And Sterility* | 2012 | 877 |
| 7 | The effects of oxidative stress on female reproduction: a review | Agarwal A | *Reproductive Biology And Endocrinology* | 2012 | 728 |
| 8 | The physiology and clinical utility of anti-Mllerian hormone in women | Dewailly D | *Human Reproduction Update* | 2014 | 540 |
| 9 | Vitamin D effects on musculoskeletal health, immunity, autoimmunity, cardiovascular disease, cancer, fertility, pregnancy, dementia and mortality-A review of recent evidence | Pludowski P | *Autoimmunity Reviews* | 2013 | 539 |
| 10 | Plastics Derived Endocrine Disruptors (BPA, DEHP and DBP) Induce Epigenetic Transgenerational Inheritance of Obesity, Reproductive Disease and Sperm Epimutations | Manikkam M | *Plos One* | 2013 | 535 |
| 11 | The prevalence and phenotypic features of polycystic ovary syndrome: a systematic review and meta-analysis | Bozdag G | *Human Reproduction* | 2016 | 535 |
| 12 | Executive Summary of the Stages of Reproductive Aging Workshop+10: Addressing the Unfinished Agenda of Staging Reproductive Aging | Harlow SD | *Journal Of Clinical Endocrinology & Metabolism* | 2012 | 534 |
| 13 | Polycystic ovary syndrome | Azziz R | *Nature Reviews Disease Primers* | 2016 | 531 |
| 14 | Polycystic ovary syndrome: definition, aetiology, diagnosis and treatment | Escobar-Morreale HF | *Nature Reviews Endocrinology* | 2018 | 515 |
| 15 | Epidemiology, diagnosis, and management of polycystic ovary syndrome | Sirmans SM | *Clinical Epidemiology* | 2014 | 501 |
| 16 | The Pathogenesis of Polycystic Ovary Syndrome (PCOS): The Hypothesis of PCOS as Functional Ovarian Hyperandrogenism Revisited | Rosenfield RL | *Endocrine Reviews* | 2016 | 496 |
| 17 | Pediatric Obesity-Assessment, Treatment, and Prevention: An Endocrine Society Clinical Practice Guideline | Styne DM | *Journal Of Clinical Endocrinology & Metabolism* | 2017 | 489 |
| 18 | Fertility and infertility: Definition and epidemiology | Vander B | *Clinical Biochemistry* | 2018 | 485 |
| 19 | Hippo signaling disruption and Akt stimulation of ovarian follicles for infertility treatment | Kawamura K | *Proceedings Of The National Academy Of Sciences Of The United States Of America* | 2013 | 453 |
| 20 | Clinical Relevance of Biomarkers of Oxidative Stress | Frijhoff J | *Antioxidants & Redox Signaling* | 2015 | 412 |
